# Synthesis of Novel Structural Hybrids between Aza-Heterocycles and Azelaic Acid Moiety with a Specific Activity on Osteosarcoma Cells

**DOI:** 10.3390/molecules25020404

**Published:** 2020-01-18

**Authors:** Gabriele Micheletti, Natalia Calonghi, Giovanna Farruggia, Elena Strocchi, Vincenzo Palmacci, Dario Telese, Silvia Bordoni, Giulia Frisco, Carla Boga

**Affiliations:** 1 Department of Industrial Chemistry ‘Toso Montanari’, Alma Mater Studiorum University of Bologna Viale Del Risorgimento, 4 402136 Bologna, Italy; elena.strocchi@unibo.it (E.S.); vincenzo.palmacci@studio.unibo.it (V.P.); dario.telese2@unibo.it (D.T.); silvia.bordoni@unibo.it (S.B.); carla.boga@unibo.it (C.B.); 2 Department of Pharmacy and Biotechnology, University of Bologna, Via Irnerio 48,40126 Bologna, Italy; giovanna.farruggia@unibo.it (G.F.); giulia.frisco2@unibo.it (G.F.); 3 National Institute of Biostructures and Biosystems, Viale delle Medaglie d’Oro, 305, 00136 Rome, Italy

**Keywords:** cancer, pyridine, pyrimidine, 9-hydroxystearic acid, azelaic acid, osteosarcoma, molecular docking

## Abstract

Nine compounds bearing pyridinyl (or piperidinyl, benzimidazolyl, benzotriazolyl) groups bound to an azelayl moiety through an amide bond were synthesized. The structural analogy with some histone deacetylase inhibitors inspired their syntheses, seeking new selective histone deacetylase inhibitors (HDACi). The azelayl moiety recalls part of 9-hydroxystearic acid, a cellular lipid showing antiproliferative activity toward cancer cells with HDAC as a molecular target. Azelayl derivatives bound to a benzothiazolyl moiety further proved to be active as HDACi. The novel compounds were tested on a panel of both normal and tumor cell lines. Non-specific induction of cytotoxicity was observed in the normal cell line, while three of them induced a biological effect only on the osteosarcoma (U2OS) cell line. One of them induced a change in nuclear shape and size. Cell-cycle alterations are associated with post-transcriptional modification of both H2/H3 and H4 histones. In line with recent studies, revealing unexpected HDAC7 function in osteoclasts, molecular docking studies on the active molecules predicted their proneness to interact with HDAC7. By reducing side effects associated with the action of the first-generation inhibitors, the herein reported compounds, thus, sound promising as selective HDACi.

## 1. Introduction

**Figure 1:**
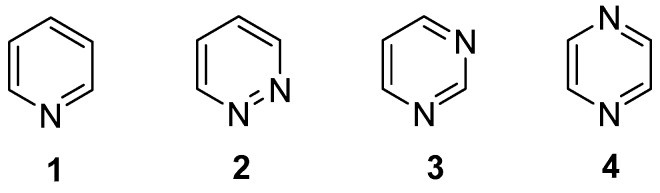
Structures of pyridine (**1**), pyridazine (**2**), pyrimidine (**3**), and pyrazine (**4**).

Nitrogen-containing heterocycles are an important class of compounds of wide interest toward many applied fields, such as material, agrochemical, and medicinal chemistry. One of the simplest aza-heteroaromatics is pyridine (**1**, Figure 1), first isolated by Anderson in 1851 [1], which plays a key role both in organic chemistry (as solvent and reactant) and in biological systems, since its nucleus belongs to many coenzymes and vitamins.

Insertion of a further aza-moiety within the six-membered pyridine structure gives 1,2-diazines, 1,3-diazines, or 1,4-diazines, whose ancestors are pyridazine (**2**), pyrimidine (**3**), and pyrazine (**4**), respectively (Figure 1).

**Figure 2:**
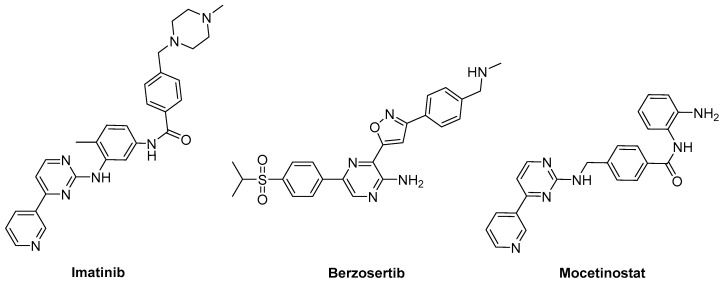
Structures of imatinib, mocetinostat, and berzosertib.

Most of them are found in plants, animals, insects, marine organisms, and microorganisms. The effectiveness in the biological field lies in the key-core structure of nucleotides and coenzymes and in their role as pharmaceutical drugs, including anticancer agents. Some examples (Figure 2) are (i) imitanib, a tyrosine kinase inhibitor used in some kinds of chronic myelogenous leukemia (CML), acute lymphocytic leukemia (ALL), and gastrointestinal stromal tumors (GIST) [2,3]; (ii) mocetinostat, a histone deacetylase inhibitor for the treatment of a variety of cancers, including follicular lymphoma, Hodgkin’s lymphoma, and acute myelogenous leukemia [4]; (iii) berzosertib (VE-822), an ATR kinase inhibitor, which proved to be effective in vivo in the treatment of non-small-cell lung and pancreatic cancer [5,6].

Pentatomic-aza-heteroaromatics bearing two different heteroatoms, such as thiazoles or imidazoles, together with their benzocondensated derivatives, are also found in many natural compounds and are of great importance as bioactive compounds [7,8]. For a long time, our interest focused on the synthesis and reactivity of thiazoles and benzothiazole derivatives [9,10,11,12,13,14,15]. Recently, we performed a S_N_Ar reaction between 2-aminobenzothiazole derivatives and 7-chloro-4,6-dinitrobenzofuroxan, and we exploited the action of the products toward the natural strain in *Vibrio* genus and different bacterial lux-biosensors [16]. Furthermore, we explored the activity shown by the azelayl scaffold connected to the 2-aminobenzothiazolyl moiety, disclosing that some of the synthesized hybrid systems behave as histone deacetylase inhibitors (HDACi) [17]. The choice to bind the benzothiazolyl group to the –(CH_2_)_7_COOMe chain through an amide bond was inspired by the following circumstances: (i) the above reported carbon chain constitutes a moiety of the endogenous cellular lipid 9-hydroxystearic acid (9-HSA) [18], with antiproliferative activity against cancer cells, including human colon cancer [19,20,21] and osteosarcoma [22,23]; (ii) 9-HSA, as well as the methyl ester [24], acts as a histone deacetylase inhibitor (HDACi) [25,26,27]; (iii) the structure of the designed compounds is analogous to the well-known vorinostat molecule [28], where a methyl ester replaced a hydroxamic acid group. Based on the above considerations, we planned to synthesize similar novel derivatives with an azelayl scaffold bound through an amide bond to pyridine, 1,3-diazine, benzimidazol, and benzotriazol moieties. All the novel compounds were tested on five cell lines. For the compounds that showed promising half maximal inhibitory concentration (IC_50_) values, further experiments and in silico studies were run to predict whether the molecular target might be HDACi, as in the case of benzothiazolyl derivatives. Herein, we report the results obtained. 

## 2. Results and Discussion

### 2.1. Chemistry

**Scheme 1:**
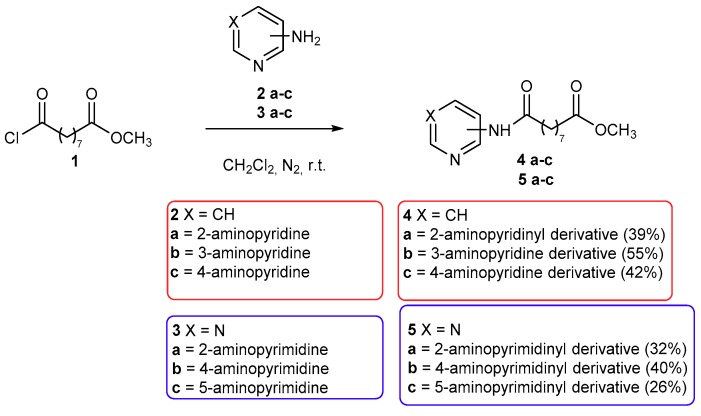
Reactions of methyl 9-chloro-9-oxononanoate (**1**) and aminopyridines **2a**–**c** or aminopyrimidines **3a**–**c**.

The two series of novel heterocyclic derivatives **4a**–**c** and **5a**–**c** were synthesized through a Schotten Bauman type reaction (Scheme 1), by reacting acyl chloride of the mono methyl azelate (**1**, synthesized from oxalyl chloride and mono methyl ester of azelaic acid) and aminopyridines **2a**–**c** or aminopyrimidines **3a**–**c** (Scheme 1). 

The reactions were carried out in anhydrous dichloromethane under nitrogen atmosphere, by using two equivalents of amine reagent to remove the hydrochloric acid formed during the reaction course. All products were purified on a silica gel column and fully characterized. They were recovered in not optimized yields ranging from 20% to 55%; in some cases, the mono methyl azelate was recovered, likely due to a certain amount of hydrolyzed acyl chloride before the amidation reaction.

**Scheme 2:**
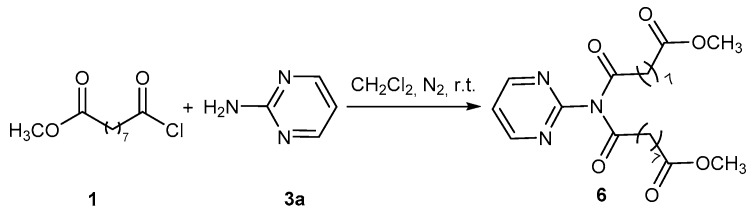
Double attack of methyl 9-chloro-9-oxononanoate (**1**) to 2-aminopyrimidine **3a**.

Concerning the reaction with 2-aminopyrimidine (**3a**), it is worth noting that, in addition to the mono acyl derivative **5a**, product **6** is formed from the attack of the amino group of **3a** on two molecules of acyl chloride **1** (Scheme 2).

To the best of our knowledge, such a reaction is not reported in the literature so far. This might be due to the stronger basicity of the amino group of the 2-aminopyrimidine (**3a**) with respect to that of the other isomers, as supported by comparing the pKa values of **3a** and **3c** (20.5 and 18.4, respectively) [29].

**Scheme 3:**
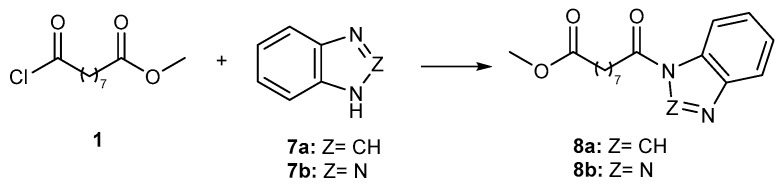
Synthesis of benzimidazole and benzotriazole derivatives **8a** and **8b**.

Adopting the same strategy, the by-reactions between **1** and benzimidazole (**7a**) or benzotriazole (**7b**) afford the azelaic derivatives **8a** and **8b**, respectively (Scheme 3).

All the above compounds underwent biological tests to assess their activity toward four cancer cells lines, U2OS (human osteosarcoma), HT29 (human colon adenocarcinoma), PC3 (human prostatic carcinoma), and IGROV1 (human ovarian carcinoma), as well as a normal human adult fibroblast cell line (see Section 2.2).

### 2.2. Biological Activity

#### 2.2.1. In Vitro Effects on Cell Viability

Cell lines included in the evaluation of toxicity profiles were malignant U2OS, HT29, PC3, and IGROV1, and a normal human adult fibroblast cell line HDFa. IC_50_ values of the drugs were calculated using Prism, fitted by means of sigmoidal fit and listed in Table 1.

IC_50_ analysis showed that all compounds had no effect on the normal cell line. Among tumor cell lines, only U2OS was sensitive to some compounds, such as **5a, 6**, and **8a**.

The chemotherapeutic drugs used in the treatment of osteosarcoma belong to two different classes: the DNA intercalating agents and the histone deacetylase inhibitors. Cisplatin, doxorubicin, and 5-fluorouracile show an IC_50_ of 1.67 µM, 0.5 µM, and 0.3 µM, respectively, in the U2OS cell line [30,31]. These effective and powerful compounds are associated with two important problems: (1) their mode of action is linked to their ability to crosslink with the purine bases on the DNA, thereby interfering with DNA repair mechanisms, causing DNA damage, and subsequently inducing death in cancer and normal cells; (2) they frequently induce multidrug resistance.

Some studies demonstrated that HDACIs, such as SAHA (SuberAniloHydroxamic Acid), sodium butyrate, and trichostatin A (TSA), are able to inhibit human osteosarcoma growth at different IC_50_ values of 5 μM, 5 μM, and 0.5 μM, respectively [32,33]. Our compounds show higher IC_50_, but they offer the important advantage of not inducing cytotoxic effects in normal cells. 

#### 2.2.2. Effects on Cell Proliferation

**Figure 3:**
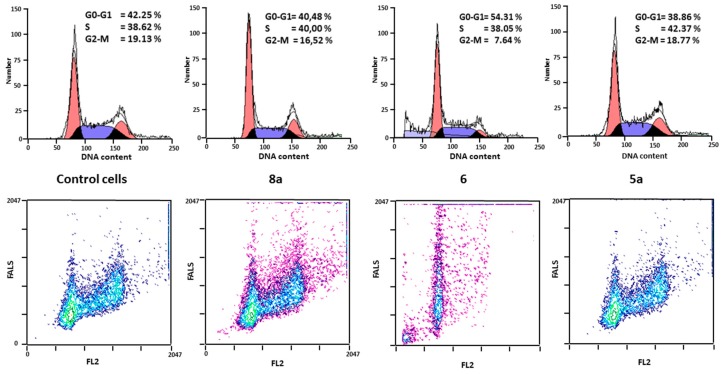
Flow cytometric assays of U2OS cells treated with compounds **5a, 6,** and **8a**, at the concentrations reported. In the upper row, the DNA distribution of a typical experiment in control and treated cells; the lower row indicates the biparametric cytograms of the DNA distribution (FL2) versus forward scatter (FAL signal. The compounds analyzed are reported between the two rows.

To gain some insight into the biological effects of these novel derivatives, the most active compounds on U2OS cell lines, 5a, **6**, and 8a were subjected to additional studies. To infer whether their effects were due to interference with cell-cycle progression, DNA profiles of cultured cells were examined by flow cytometry. While compound **5a** and **8a** caused a slight accumulation in the synthesis (S) phase, compound **6** induced an increase in the gap 0/gap 1 (G0/G1) fraction, with concomitant decrease in the G2/mitosis (M) phase. Interestingly, compound **6** enlarged cell nucleus size, as shown in Figure 3, where the propidium iodide (PI) fluorescence, indicating the amount of DNA per cell, is plotted against the forward scatter (FS) signal, which is proportional to the size of the analyzed particle. Again, the mean channel of FS distribution was very similar for control cells, **5a**, and **8a** treated samples (730, 735, and 780, respectively), while sample **6** showed a mean channel of the FS distribution of 905. As clear from Figure 3, the nuclei in all cell-cycle phases were affected.

Nuclear size and shape are currently not only considered connected to the changes in DNA content and structure during cell-cycle progression, but it is also well known that alteration of nuclear morphology could be due to differentiation [34], pathologies as cancer [35,36], or to senescence [37].

Nuclear size could also be affected by drugs, such as intercalating drugs [37] or etoposide [38]. Interestingly, the fatty palmitic acid increases nuclear size in chick embryonic cardiomyocytes [39], as well as in mouse fibroblasts [40]. The mechanism of these effects is not disclosed, even if it was hypothesized that the lipid, or its metabolites, could interact with DNA, thereby altering the structure of the nucleus, or that they activate cellular metabolism, thereby increasing protein synthesis [39].

#### 2.2.3. Effect on Histone Acetylation

**Figure 4:**
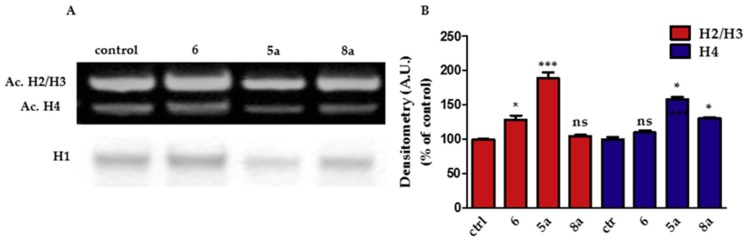
Effect of compounds **5a**, **6**, and **8a** on histone acetylation levels. (**A**) Cell nuclear extracts were prepared and subjected to Western blot analysis for acetylated (Ac) histones H2/H3 and H4. Histone H1 was used as loading control. A representative experiment is shown, which was repeated three times. (**B**) Densitometric analysis of the bands (mean ± SD; *n* = 3) is shown. * *p* ≤ 0.05, *** *p* ≤ 0.01 vs. control.

To identify acetylated histones, we analyzed the nuclear cell lysates by Western blot using a 15% polyacrylamide gel electrophoresis. After electrophoresis, the proteins were transferred to a nitrocellulose membrane and then immunoblotted with an anti-acetyl lysine monoclonal antibody. As shown in Figure 4, the antibody could detect the accumulation of acetylated proteins induced in U2OS cells by treatment with compounds **6**, **5a**, and **8a**. Differences in the density of bands are thought to reflect differences in protein acetylation levels. Histone acetylation signals were quantified by densitometry and normalized on histone H1. Histones H2/H3 acetylation increased by 28% upon treatment with **6** for 6 h with no effect on H4 acetylation, whereas compound **8a** induced hyperacetylation by 30% on histone H4 only. Interestingly, compound **5a** caused a post-transcriptional modification of both H2/H3 and H4 histones, increasing acetylation by 89% and 22%, respectively.

**Figure 5:**
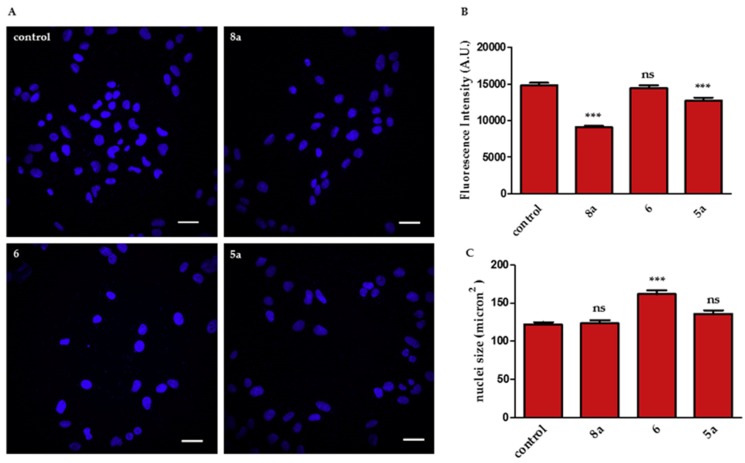
Confocal microscopy of Hoechst 33,342 nuclear staining in control and treated U2OS cells. (**A**) U2OS cells were treated with compounds **5a**, **6**, or **8a** for 24 h, and then stained with Hoechst 33,342. Representative images are shown (scale bar = 20 μm). (**B**) Image densitometry measured using ImageJ (*** *p* < 0.01 with respect to control). (**C**) Cell size was estimated by Hoechst 33,342 staining by image densitometry using ImageJ (*** *p* < 0.01 with respect to control).

These effects induced by the above compounds on histone acetylation are not associated with events involved in apoptotic death. The analysis of the nucleus labeling with Hoechst 33,342 shows indeed no morphological alterations typical of apoptotic cell death (Figure 5). However, the compounds induced a state of nuclear alteration, as shown in Figure 5, where it is possible to see how the compounds **5a** and **8a** caused a dim staining of nuclei, while compound **6** induced an increase in nuclear size, as observed by the flow cytometric assay. 

### 2.3. Docking Evaluation

To verify the biochemical data observed for **5a**, **6**, and **8a** species and to explore the binding mode and the potential affinity toward the catalytic Zn^2+^ active site of some HDACs, the synthesized inhibitors were docked to the following structures involved in the histone deacetylation pathway: (*i*) HDAC1, HDAC2, HDAC3, and HDAC8, belonging to the class I HDACs; (*ii*) HDAC4 and HDAC7, belonging to the class II HDACs. 

The target macromolecules were selected by analyzing the action modes of some antitumor drugs pertaining to the category of HDACs. Recent studies revealed an unexpected function for HDAC7 in osteoclasts different from the function of HDAC3. Suppression of HDAC7 enhances osteoclast formation, whereas overexpression of HDAC7 impairs osteoclast formation, indicating that HDAC7 represses osteoclast differentiation [41]. Moreover, HDAC7 was found to be overexpressed in pancreatic cancer and acute lymphoblastic leukemia and was reported as insensitive to its previously designated HDACi, trichostatin A (TSA) [42,43].

Our benzimidazolyl (**8a**) and pyrimidinyl (**5a**,**6**) derivatives were found to have the minimum requirements for tight binding at the pocket Zn^2+^ active site of the HDAC domain whether in class I or II examined macromolecules. In the literature, compounds similar to the studies herein showed evidence for Zn complex formation [44], and the selected target proteins for docking evaluation of the proposed compounds were retrieved from the Protein Data Bank (PDB; http:/www.rcsb.org/pdb/) and were as follows: HDAC1 (PDB: 5ICN), HDAC2 (PDB: 4LXZ), HDAC3 (PDB: 4A69, HDAC8 (PDB: 4QA3), HDAC4 (PDB: 2VQM), and HDAC7 (PDB: 3C0Z). Binding modes and binding affinities of the evaluated compounds within the catalytic Zn^2+^ pocket site of all the selected HDACs were calculated using the Autogrid 4.0 and Autodock 4.2 programs [45]. The ADT 1.6.1 package was used for visualizing the results (for the preparation of the molecules and the target macromolecules, and for the procedure of molecular docking, see Section 3).

#### Analysis of the Binding Mode

The binding pose of the evaluated compounds and their interactions in the active binding site of the selected HDACs were analyzed. The predicted binding free energy (BE, which includes intermolecular energy and torsional free energy) was used as a ranking criterion. The conformation with the lowest ranking docked binding energy (BE) was considered as the “best” docking result.

Satisfactory docking results with good affinity results were observed for the three compounds (**5a, 6**, and **8a**) whether for the evaluated class I or class II HDACs, as well as virtual constants of inhibition (*K*i) at micromolar concentration. The compounds docked and perfectly fitted into the active pocket site of the examined HDAC2, HDAC8, and HDAC7, as indicated from binding free energy reported in Table 2 and from analysis of the ligand binding pose inside the active binding sites.

Along the evaluated compounds, the best docking results were observed for the class I HDAC8 and class II HDAC7; the best poses of compounds **5a**, **6**, and **8a** into the active binding site had an estimated binding free energy (∆G) of −8.05, −8.14, and −8.63 kcal/mol, respectively (for HDAC8), and −7.26, −7.00, and −7.61 kcal/mol, respectively (for HDAC7). The best calculated corresponding inhibition constants (*K*i) related to HDAC8 were found to be 1.26 μM, 1.07 μM, and 472.15 nM, respectively, for **5a**, **6**, and **8a** compounds, while they were 4.78 μM, 7.37 μM, and 2.62 μM, for the same compounds in the case of HDAC7. Similar results for ∆G of −7.24 and −7.73 kcal/mol were observed with HDAC2 for compounds **6** and **8a** with the corresponding best calculated inhibition constants (*K*i) equal to 4.94 μM and 2.17 μM, respectively. Compound **6** also showed similarly good results with the other examined HDAC4 (∆G of −7.61 kcal/mol and inhibition constant (*K*i) equal to 2.62 μM).

The structures of HDAC enzymes are characterized by the active Zn^2+^ ion at the pocket bottom, the hydrophobic channel reaching the active Zn^2+^ ion, and the surface rim at the entrance of the pocket. Thus, to assess the precise binding pose of the synthesized compounds in the binding pocket of HDAC1 (PDB code: 5ICN), HDAC2 (PDB code 4LXZ), HDAC3 (PDB code 4A69), HDAC8 (PDB code 4QA3), HDAC4 (PDB code 2VQM), and HDAC7 (PDB code: 3C0Z), we performed a molecular docking study. 

This docking study acknowledged the inhibitory potential of **5a, 6**, and **8a** against both HDAC8 and HDAC7 isoforms. 

The docking results showed that these inhibitors are able to coordinate to the zinc ion. The in silico docking study displayed different poses of synthesized compounds for HDAC8 and HDAC7 isoforms.

All the compounds (**5a, 6**, and **8a)** are characterized by one (**5a** and **8a**) or two (**6**) terminal methyl ester groups at the end of the long aliphatic chain, connected to the heterocyclic moiety by an amide bond, and they exhibit slightly better values of binding free energies to class I HDAC8, (Table 2). 

**Figure 6:**
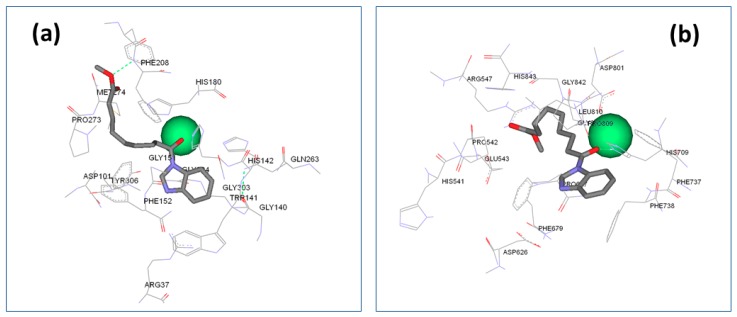
Molecular docking analysis of compound **8a** (showing best *K*i inhibition docking results) with HDAC8 and HDAC7 isoforms. Docking poses of compound **8a** in the binding site of (**a**) HDAC8 structure (Protein Data Bank identifier (PDB ID): 4QA3) and (**b**) HDAC7 structure (PDB ID: 3C0Z). In both pictures, the zinc ion is represented as a green sphere**.**

For the sake of clarity, in Figure 6, we report only the docking pose of the molecule **8a** (showing the best *K*i inhibition) in the binding site of HDAC8 and HDAC7, belonging to class I and II HDACs. In the Supplementary Materials, the figures related to the following docking poses are reported: for molecule **8a** in HDAC2 (Figure S28), for molecules **5a** and **6** in the binding site of HDAC8, HADC7, and HDAC2 (Figures S29 and S30, respectively), and for all the evaluated compounds in the HDAC8 and HDAC7 active binding sites (Figure S31).

The terminal ester group of compound **8a** and of one lateral chain of **6** were accommodated into the active pocket found in the HDAC7 isoform, while **5a** had the pyrimidine group inserted in the bottom of the pocket. The benzimidazole moiety of **8a** and the pyrimidinyl group of compound **5a** fit perfectly into the active pocket in HDAC8 differently from the HDAC7 isoform, while the orientation of compound **6** showed a terminal ester group of one lateral chain into the pocket. The docking pose of **5a**, **6**, and **8a** with HDAC8 indicated that the oxygen atom of the amide group of all compounds formed a potential hydrogen bonding interaction with the NH^+^ of His180 at the rim of the pocket.

On the other hand, the pose of all the compounds to HDAC7 indicated that the oxygen atom of the amide group formed a potential hydrogen bonding interaction with the NH^+^ of His709 at the rim of the pocket. A comprehensive overview is reported in the Supplementary Materials (Figure S31). 

**Figure 7:**
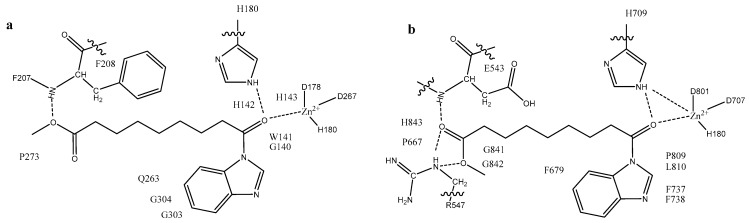
Hydrogen bond and chelation interactions in the active site of (**a**) complex **8a**–HDAC8-4QA3, and (**b**) complex **8a**–HDAC7-3C0Z. Amino-acid residues within 5 Å are indicated without a dashed line.

Figure 7 reports hydrogen bond and chelation interactions in the active site of complexes between **8a** and HDAC8-4QA3 (**a**) and **8a** with HDAC7-3C0Z (**b**); amino-acid residues within 5 Å are indicated without a dashed line.

As can be seen, the coordination sphere of the zinc ion involved, in both reported cases in Figure 7, interactions with the oxygen atom of amide group of compound **8a** and two histidine residues (one of them also involved in a hydrogen bond with the oxygen atom of the amide group, and two aspartic acid residues).

Tables S1–S3 (Supplementary Materials) report the “virtual” interactions of all compounds docked with amino-acid residues and the zinc ion in the active site of HDAC8-4QA3, HDAC7-3C0Z, and HDAC2-4LXZ.

## 3. Materials and Methods 

### 3.1. Chemical Syntheses

The reagents used, unless stated otherwise, were purchased from Sigma-Aldrich (Milan, Italy). CH_2_Cl_2_ was anhydrified by distillation over P_2_O_5_. Chromatographic purifications (FC) were carried out on glass columns packed with silica gel (Merck grade 9385, 230–400 mesh particle size, 60 Å pore size) at medium pressure. Thin-layer chromatography (TLC) was performed on silica gel 60 F254-coated aluminum foils (Fluka, Buchs, Switzerland). The spots related to 9-methoxy-9-oxononanoic acid were revealed using a bromocresol green solution (6% in ethanol). 

The nuclear magnetic resonance spectra were recorded at 25 °C on Varian spectrometers Mercury 400 or Inova 600 (Varian, Palo Alto, CA, USA) operating at 400 or 600 MHz (for ^1^H-NMR) and 100.56 or 150.80 MHz (for ^13^C-NMR), respectively. Signal multiplicities were established by DEPT-135 experiments. Chemical shifts were measured in δ (ppm) with reference to the solvent (δ= 7.26 ppm and 77.00 ppm for CDCl_3_, for ^1^H- and ^13^C-NMR, respectively). *J*-values are given in Hz. Electrospray ionization (ESI)-MS and ESI high-resolution (HR)MS spectra were recorded using a Waters ZQ 4000 and Xevo instrument, respectively. Melting points (m.p.) were measured on a Büchi 535 apparatus (Flawil, Zwitzerland) and are uncorrected.

#### 3.1.1. Synthesis of Methyl 9-Chloro-9-oxononanoate (**1**)

9-Methoxy-9-oxononanoic acid (1.12 mL, 5.87 mmol) was introduced in a dried three-necked round-bottom flask (equipped with a dropping funnel and kept under nitrogen atmosphere) and dissolved in 45 mL of anhydrous CH_2_Cl_2_. Oxalyl chloride (0.53 mL, 6.26 mmol), diluted in 5 mL of anhydrous CH_2_Cl_2_, was introduced in the funnel and added dropwise to the magnetically stirred solution over 10 min. The reaction was monitored through ^1^H-NMR spectroscopy. When the signals of the starting acid disappeared, the solvent was removed under reduced pressure, with care to avoid contact with moisture, and the residue was dissolved in anhydrous CH_2_Cl_2_, in an amount calculated in order to obtain a 0.5 M solution of acyl chloride. The chemical–physical data of compound **1** agreed with those reported in the literature [18].

#### 3.1.2. General Procedure for the Synthesis of Compounds **4a**–**c**, **5a**–**c**, **6**, and **8a,b**

In a dried apparatus and under nitrogen atmosphere, 1.0 mL of a 0.5 M solution of **1** in CH_2_Cl_2_ (0.0005 mol) was added to a magnetically stirred solution containing 0.001 mol of the selected heterocyclic compound **2a**–**c** (or **3a**–**c**, or **7a,b**) dissolved in 5 mL of anhydrous CH_2_Cl_2_. The reaction course was monitored by ^1^H-NMR spectroscopy until the acyl chloride signals disappeared. Then, water (10 mL) was added, and the mixture was extracted with dichloromethane (3 × 10 mL). The organic layer was dried over anhydrous MgSO_4_ and filtered. After removal of the solvent under reduced pressure, the products were purified by column chromatography on silica gel or by bulb-to-bulb distillation.

*Methyl 9-oxo-9-(pyridin-2-ylamino)nonanoate* (**4a**), purified by FC (ethyl acetate/dichloromethane 40/60). White solid; 0.054 g (39%); m.p. 59–60 °C; ^1^H-NMR (600 MHz, CDCl_3_), δ (ppm): 8.43 (br. s, 1H, NH), 8.25 (d, *J* = 5.2 Hz, 1 H, CH), 8.21 (d, *J* = 8.3 Hz, 1 H, CH), 7.69 (dt, *J*_1_ = 7.2 Hz, *J*_2_ = 1.7 Hz, 1H, CH), 7.02 (dd, *J*_1_ = 7.1 Hz, *J*_2_ = 5.1 Hz, 1 H, CH), 3.65 (s, 3 H, COOCH_3_), 2.37 (t, *J* = 7.6 Hz, 2 H, CH_2_CON), 2.28 (t, *J* = 7.6 Hz, 2 H, CH_2_COOCH_3_), 1.71 (quint., *J* = 7.5 Hz, 2 H, CH_2_CH_2_CON), 1.60 (quint, *J* = 7.1 Hz, 2 H, CH_2_CH_2_COOCH_3_), 1.39–1.27 (m, 6 H, CH_2_); ^13^C-NMR (150 MHz, CDCl_3_) δ (ppm): 174.2 (C), 171.8 (C), 151.5 (C), 147.6 (CH), 138.4 (CH), 119.6 (CH), 114.1 (CH), 51.4 (CH_3_), 37.6 (CH_2_), 34.0 (CH_2_), 28.94 (CH_2_), 28.9 (CH_2_), 28.8 (CH_2_), 25.2 (CH_2_), 24.8 (CH_2_); ESI-MS^−^ (*m*/*z*): 313 [M + Cl]^−^; ESI-HRMS: calculated for C_15_H_22_N_2_NaO_3_^+^: 301.1523, found: 301.1528

*Methyl 9-oxo-9-(pyridin-3-ylamino)nonanoate* (**4b**), purified by FC (ethyl acetate/dichloromethane 40/60). White solid; 0.076 g (55%); m.p. 75.5–77.7 °C; ^1^H-NMR (600 MHz, CDCl_3_) δ (ppm) 8.80 (br.s, 1 H, NH); 8.58 (d, *J* = 2.3 Hz, 1 H, CH); 8.26 (d, *J* = 4.5 Hz, 1 H, CH); 8.20 (d, *J* = 8.1 Hz, 1 H, CH); 7.23 (dd, *J*_1_ = 8.2 Hz, *J*_2_ = 4.6 Hz, 1 H, CH); 3.63 (s, 3 H, COOCH_3_); 2.35 (t, *J* = 7.8 Hz, 2 H, CH_2_CON); 2.26 (t, *J* = 7.8 Hz, 2 H, CH_2_COOCH_3_); 1.67 (quint., *J* = 7.3 Hz, 2 H, CH_2_CH_2_CON); 1.56 (quint., *J* = 7.3 Hz, 2 H, CH_2_CH_2_COOCH_3_); 1.34–1.23 (m, 6 H, CH_2_);^13^C-NMR (150 MHz, CDCl_3_) δ (ppm): 174.4 (C), 172.4 (C), 144.3 (CH), 140.7 (CH), 135.5 (C), 127.4 (CH), 123.8 (CH), 51.4 (CH_3_), 37.2 (CH_2_), 33.9 (CH_2_), 28.8 (CH_2_), 28.76 (CH_2_), 28.72 (CH_2_), 25.2 (CH_2_), 24.7 (CH_2_); ESI-MS^+^ (*m*/*z*): 279 [M + H]^+^, 301 [M + Na]^+^; ESI-HRMS: calculated for C_15_H_22_N_2_NaO_3_^+^: 301.1523, found: 301.1528.

*Methyl 9-oxo-9-(pyridin-4-ylamino)nonanoate* (**4c**), purified by FC (methanol/ethyl acetate 5/95). White solid; 0.059 g (42%); m.p. 83–85 °C; ^1^H-NMR (600 MHz, CDCl_3_) δ (ppm): 8.91 (s, 1 H, NH), 8.43 (d, *J* = 5.1 Hz, 2 H, CH), 7.56 (d, *J* = 5.1 Hz, 2 H, CH), 3.64 (s, 3 H, COOCH_3_), 2.35 (t, *J* = 7.6 Hz, 2 H, CH_2_CON), 2.28 (t, *J* = 7.6 Hz, 2 H, CH_2_COOCH_3_), 1.67 (quint., *J* = 7.4 Hz, 2 H, CH_2_CH_2_CON), 1.57 (quint., *J* = 7.4 Hz, 2 H, CH_2_CH_2_COOCH_3_), 1.35–1.25 (m, 6 H, CH_2_); ^13^C-NMR (150 MHz, CDCl_3_) δ (ppm): 174.4 (C), 172.7 (C), 149.9 (CH), 146.0 (C), 113.7 (CH), 51.5 (CH_3_), 37.5 (CH_2_), 33.9 (CH_2_), 28.8 (CH_2_), 28.77 (CH_2_), 28.73 (CH_2_), 25.1 (CH_2_), 24.7 (CH_2_); ESI-MS^−^ (*m*/*z*): 277 [M − H]^−^, 313 [M + Cl]^−^; ESI-HRMS: calculated for C_15_H_22_N_2_NaO_3_^+^: 301.1523, found: 301.1528.

*Methyl 9-oxo-9-(pyrimidin-2-ylamino)nonanoate* (**5a**), purified by FC (methanol/ethyl acetate 5/95). White solid; yield 0.067 g (32%); m.p. 86.2–87.3 °C; ^1^H-NMR (400 MHz, CDCl_3_) δ (ppm): 9.55 (br.s, 1 H, NH), 8.61 (d, *J* = 4.9 Hz, 2 H, CH), 6.97 (7, *J* = 4.9 Hz, 1 H, CH) 3.62 (s, 3 H, COOCH_3_), 2.73 (t, *J* = 7.4 Hz, 2 H, CH_2_CON), 2.26 (t, *J* = 7.6 Hz, 2 H, CH_2_COOCH_3_), 1.70 (quint., *J* = 7.3 Hz, 2 H, CH_2_CH_2_CON), 1.59 (quint., *J* = 7.0 Hz, 2 H, CH_2_CH_2_COOCH_3_), 1.45–1.2 (m, 6H, CH_2_); ^13^C-NMR (100 MHz, CDCl_3_) δ (ppm): 174.2 (C), 173.7 (C), 158.2 (CH), 157.6 (C), 115.9 (CH), 51.3 (CH_3_), 37.3 (CH_2_), 33.9 (CH_2_), 28.93 (CH_2_), 28.90 (CH_2_), 28.8 (CH_2_), 24.8 (CH_2_) (one signal overlapped); ESI-MS^+^ (*m*/*z*): 280 [M + H]^+^, 302 [M + Na]^+^, 318 [M + K]^+^; ESI-HRMS: calculated for C_14_H_22_N_3_NaO_3_^+^: 302.1475, found: 302.1481.

*Methyl 9-oxo-9-(pyrimidin-4-ylamino)nonanoate* (**5b**), purified by FC (methanol/ethyl acetate 5/95). White solid; 0.056 g (40%); m.p. 92.0–94.2 °C; ^1^H-NMR (600 MHz, CDCl_3_) δ (ppm): 8.84 (s, 1 H, CH), 8.62 (d, *J* = 5.7 Hz, 1 H, CH), 8.18 (dd, *J*_1_ = 5.9 Hz, *J*_2_ = 1.2 Hz, 1 H, CH), 8.13 (br.s, 1 H, NH), 3.66 (s, 3 H, COOCH_3_), 2.42 (t, *J* = 7.5 Hz, 2 H, CH_2_CON), 2.30 (t, *J* = 7.2 Hz, 2 H, CH_2_COOCH_3_), 1.72 (quint., *J* = 7.2 Hz, 2 H, CH_2_CH_2_CON), 1.62 (quint., *J* = 7.1 Hz, 2 H, CH_2_CH_2_COOCH_3_), 1.42–1.24 (m, 6 H, CH_2_); ^13^C-NMR (150 MHz, CDCl_3_) δ (ppm): 174.2 (C), 172.5 (C), 158.4 (CH), 158.2 (CH), 156.9 (C), 110.2 (CH), 51.5 (CH_3_), 37.7 (CH_2_), 34.0 (CH_2_), 28.9 (CH_2_), 28.8 (CH_2_), 24.9 (CH_2_), 24.8 (CH_2_) (one signal overlapped); ESI-MS^+^ (*m*/*z*): 280 [M + H]^+^, 302 [M + Na]^+^, 318 [M + K]^+^; ESI-HRMS: calculated for C_14_H_22_N_3_NaO_3_^+^: 302.1475, found: 302.1481.

*Methyl 9-oxo-9-(pyrimidin-5-ylamino)nonanoate* (**5c**), purified by FC (methanol/ethyl acetate 5/95). Orange solid; 0.036 g (26%); m.p. 78.6–80.2 °C; ^1^H-NMR (300 MHz, CDCl_3_) δ (ppm) 9.29 (s, 2 H, CH), 8.98 (s, 1 H, CH); 8.63 (br. s, 1 H, NH), 3.66 (s, 3 H, COOCH_3_), 2.47 (t, *J* = 7.6 Hz, 2 H, CH_2_CON), 2.31 (t, *J* = 7.3 Hz, 2 H, CH_2_COOCH_3_), 1.74 (quint., *J* = 7.4 Hz, 2 H, CH_2_CH_2_CON), 1.62 (quint., *J* = 7.4 Hz, 2H, CH_2_CH_2_COOCH_3_), 1.42–1.28 (m, 6 H, CH_2_); ^13^C-NMR (150 MHz, CDCl_3_) δ (ppm): 174.4 (C), 172.5 (C), 150.7 (CH), 147.2 (CH), 134.8 (C), 51.5 (CH_3_), 37.1 (CH_2_), 34.0 (CH_2_), 28.7 (CH_2_), 25.0 (CH_2_), 24.7 (CH_2_) (two signals overlapped); ESI-MS^+^ (*m*/*z*): 280 [M + H^+^]^+^, 302 [M + Na^+^]^+^, 318 [M + K^+^]^+^; ESI-HRMS: calculated for C_14_H_22_N_3_NaO_3_^+^: 302.1475, found: 302.1481.

*Dimethyl 9,9’-(pyrimidin-2-ylazanediyl)bis(9-oxononanoate)* (**6**), purified by FC (methanol/ethyl acetate 5/95). Colorless liquid; 0.016 g (14%); ^1^H-NMR (600 MHz, CDCl_3_) δ (ppm): 8.81 (d, *J* = 4.7 Hz, 2 H, CH), 7.33 (t, *J* = 4.8 Hz, 1 H, CH), 3.59 (s, 6 H, COOCH_3_), 2.47 (t, *J* = 7.4 Hz, 4 H, CH_2_CON), 2.22 (t, *J* = 7.6 Hz, 4 H, CH_2_COOCH_3_), 1.60–1.48 (m, 8 H, CH_2_CH_2_COOCH_3_), 1.29–1.18 (m, 12 H, CH_2_), ^13^C-NMR (150 MHz, CDCl_3_) δ (ppm): 175.0 (C), 174.0 (C), 159.5 (C), 159.3 (CH), 120.3 (CH), 51.2 (CH_3_), 37.9 (CH_2_), 33.8 (CH_2_), 28.75 (CH_2_), 28.70 (CH_2_), 28.66 (CH_2_), 28.60 (CH_2_), 24.6 (CH_2_), 24.0 (CH_2_); ESI-MS^−^ (*m*/*z*): 498 [M + Cl]^−^; ESI-HRMS: calculated for C_24_H_37_N_3_NaO_6_^+^: 486.2575, found: 486.2580.

*Methyl 9-(1H-benzo[d]imidazol-1-yl)-9-oxononanoat*e (**8a**), purified by bulb-to-bulb distillation at 150 °C and 0.1 mmHg. White solid; 0.075 g (50%); m.p. 83.0–84.3 °C; ^1^H-NMR (600 MHz, CDCl_3_) δ (ppm): 8.40 (s, 1 H, CH), 8.25 (d, *J* = 7.9 Hz, 1 H, CH), 7.80 (d, *J* = 7.0 Hz, 1 H, CH), 7.43 (dt, *J*_1_ = 8.5 Hz, *J*_2_ = 1.2 Hz, 1 H, CH), 7.40 (dt, *J*_1_ = 9.3 Hz, *J*_2_ = 1.6 Hz, 1 H, CH), 3.67 (s, 3 H, COOCH_3_), 3.00 (t, *J* = 7.4 Hz, 2 H, CH_2_CON), 2.32 (t, *J* = 7.3 Hz, 2H, CH_2_COOCH_3_), 1.88 (quint., *J* = 7.8 Hz, 2 H, CH_2_CH_2_CON), 1.64 (quint., *J* = 7.8 Hz, 2 H, CH_2_CH_2_COOCH_3_), 1.47 (quint., *J* = 7.7 Hz, 2H, CH_2_), 1.43–1.32 (m, 4 H, CH_2_); ^13^C-NMR (150 MHz, CDCl_3_) δ (ppm): 174.2 (C), 170.3 (C), 143.9 (CH), 140.9 (C), 131.5 (C), 125.9 (CH), 125.0 (CH), 120.5 (CH), 115.6 (CH), 51.5 (CH_3_), 35.9 (CH_2_), 34.0 (CH_2_), 28.9 (CH_2_), 28.86 (CH_2_), 28.84 (CH_2_), 24.8 (CH_2_), 24.2 (CH_2_); ESI-MS^+^ (*m*/*z*): 303 [M + H^+^]^+^, 325 [M + Na^+^]^+^; ESI-HRMS: calculated for C_17_H_22_N_2_NaO_3_^+^: 325.1523, Found: 325.1528

*Methyl 9-(1H-benzo[d][1,2,3]triazol-1-yl)-9-oxononanoate* (**8b**), purified by bulb-to-bulb distillation at 150 °C and 0.1 mmHg. White solid; 0.032 g (20%); m.p: 55.5–57.2 °C; ^1^H-NMR (600 MHz, CDCl_3_) δ (ppm): 8.28 (d, *J* = 8.3 Hz, 1 H, CH), 8.10 (d, *J* = 8.0 Hz, 1 H, CH), 7.64 (t, *J* = 7.8 Hz, 1 H, CH), 7.49 (t, *J* = 7.8 Hz, 1 H, CH), 3.65 (s, 3 H, COOCH_3_), 3.40 (t, *J* = 7.4 Hz, 2 H, CH_2_CON), 2.30 (t, *J* = 7.6 Hz, 2 H, CH_2_COOCH_3_), 1.89 (quint., *J* = 7.8 Hz, 2 H, CH_2_CH_2_CON), 1.63 (quint., *J* = 7.5 Hz, 2 H, CH_2_CH_2_COOCH_3_), 1.48 (quint., *J* = 7.7 Hz, 2 H, CH_2_), 1.42–1.32 (m, 4 H, CH_2_); ^13^C-NMR (150 MHz, CDCl_3_) δ (ppm) 174.1 (C), 172.5 (C), 146.1 (C), 131.1 (C), 130.3 (CH), 126.0 (CH), 120.1 (CH), 114.4 (CH), 51.4 (CH_3_), 35.4 (CH_2_), 34.0 (CH_2_), 28.9 (CH_2_), 28.8 (CH_2_), 24.8 (CH_2_), 24.3 (CH_2_) (one signal overlapped); ESI-MS^+^ (*m*/*z*): 304 [M + H^+^]^+^, 326 [M + Na^+^]^+^.

### 3.2. Cell Culture and Treatments

#### 3.2.1. Cell Culture

The human prostate cancer (PC3), human colon cancer (HT29), human bone osteosarcoma (U2OS), and normal human adult fibroblast (HDFa) cell lines were purchased from American Type Culture Collection (ATCC, Manassas, VA, USA), while the human ovarian cancer cell line (IGROV1) was kindly provided by Istituto Nazionale Tumori (IRCCS, Milano, Italy). Cells were cultured in Roswell Park Memorial Institute (RPMI)-1640 medium (Labtek Eurobio, Milan, Italy), supplemented with 10% fetal calf serum (FCS; Euroclone, Milano, Italy) and 2mM l-glutamine (Sigma-Aldrich, Milano, Italy), at 37 °C and 5% CO_2_ atmosphere. The compounds were dissolved in dimethyl sulfoxide (DMSO) in a 60 mM stock solution. In cell treatments, the final DMSO concentration never exceeded 0.1%.

#### 3.2.2. MTT (3-(4,5-dimethylthiazol-2-yl)-2,5-diphenyltetrazolium bromide) Assay

U2OS were seeded at 1.5 × 10^4^ cells/well in a 96-well culture plastic plate (Orange Scientific, Braine-l’Alleud, Belgium) and, after 24 h of growth, cells were exposed for additional 48 h to increasing concentrations of compounds (0.1 µM and 500 µM) solubilized in RPMI-1640 medium. On the day of measurement, the culture medium was replaced with 0.1 mL of 3-(4,5-dimethylthiazolyl-2)-2,5-diphenyltetrazolium bromide (MTT, Sigma-Aldrich) dissolved in phosphate-buffered saline (PBS) at the concentration of 0.2 mg/mL, and samples were incubated for 2 h at 37 °C. To dissolve the blue-violet formazan salt crystals formed, 0.1 mL of isopropyl alcohol was added to each well and incubated for 20 min. The absorbance at 570 nm was measured using a multiwell plate reader (Wallac Victor2, PerkinElmer, Milano, Italia), and viability was compared with that of untreated cells, used as controls.

#### 3.2.3. Cell Cycles

For the cell-cycle assay, U2OS cells were treated for 24 h with 35 μM of compound **6**, 50 μM of **5a**, or 50 μM of **8a**, detached with 0.11% trypsin (Sigma-Aldrich, S.Louis, MO, USA)/0.02% ethylenediaminetetraacetic acid (EDTA) (Sigma-Aldrich, S.Louis, MO, USA), washed in PBS, and centrifuged. The pellet was resuspended in 0.01% Nonidet P-40 (Sigma-Aldrich, S.Louis, MO, USA), 10 μg/mL RNase (Sigma-Aldrich, S.Louis, MO), 0.1% sodium citrate, and 50 μg/mL propidium iodide (PI) (Sigma-Aldrich, S.Louis, MO, USA), for 30 min at room temperature in the dark. PI fluorescence and forward scatter (FS) signals were analyzed using a Beckman Coulter Epics XL-MCL flow cytometer. DNA distribution in the cell cycle was analyzed by the MODFIT 5.0 software. As the signal collected by a multichannel analyzer, the mean of the FS distribution is expressed as mean channel.

#### 3.2.4. Histone Extraction, SDS-PAGE, and Western Blot

U2OS were cultured with **5a**, **6**, or **8a**, for 6 h, and the histone fraction was immediately extracted. Cells were harvested using 0.11% trypsin and 0.02% EDTA, washed twice with 10 mM sodium butyrate (NaBu) in PBS, and nuclei were isolated according to Amellem et al. [46]. The nuclear pellet was suspended in 0.1 mL of ice-cold water using a Vortex mixer, and concentrated H_2_SO_4_ was added to the suspension to give a final concentration of 0.4 N. After incubation at 4 °C for 1 h, the suspension was centrifuged for 5 min at 14,000× *g*, and the supernatant was taken and mixed with 1 mL of acetone. After overnight incubation at −20 °C, the coagulate material was collected by microcentrifugation and air-dried, and proteins were quantified using a protein assay kit (Bio-Rad, Hercules, CA, USA). Histones were analyzed as previously described [24]. Briefly, histones were resolved by 15% SDS-PAGE and immunoblotted with anti-acetylated lysine antibody (Cell Signaling Technology, Beverly, MA, USA); the detection of immunoreactive bands was performed with a secondary antibody conjugated with horseradish peroxidase and developed with an enhanced chemiluminescence (ECL) system, developed with the enhanced chemiluminescence system Clarity Western (Bio-Rad, Hercules, CA, USA), and quantification was done by Fluor-S Max MultiImager (Bio-Rad). Histone acetylation signals were quantified by densitometry and normalized on histone H1.

#### 3.2.5. Hoechst 33,342 Staining

Nuclear morphology was assayed using a specific dye Hoechst 33,342. U2OS treated cells were washed, fixed, and then stained with 1 µM Hoechst 33,342 (Sigma-Aldrich, St Louis, MO, USA) for 15 min. Samples were embedded in Mowiol and analyzed using a Nikon C1s confocal laser-scanning microscope, equipped with a Nikon PlanApo 40×, 1.4 numerical aperture (NA) oil immersion lens. Images were quantified by ImageJ software (IMAJ 1.x).

### 3.3. Docking

Crystal structures of target HDACs, HDAC1 (PDB: 5ICN), HDAC2 (PDB: 4LXZ), HDAC3 (PDB: 4A69, HDAC8 (PDB: 4QA3), HDAC4 (PDB: 2VQM), and HDAC7 (PDB: 3C0Z), used for the docking study, were obtained from the Protein Data Bank (http:/www.rcsb.org/pdb/). The target proteins were prepared using Maestro 9.1 software (Schrödinger, LLC, New York, NY, USA 2010) deleting waters, optimizing H-Bond assignments, and deleting original ligands. Before starting the docking calculations, ligand geometry optimization was done using Gaussian 3 [47] by semi-empirical AM1 [48] in order to obtain the minimum-energy conformation. The computational analysis was executed using AutoDock 4.2 [45] and Autogrid 4.0 on a Dual-Xeon T7400 Dell workstation. Grids (one grid for each atom type in the ligand, plus an electrostatic and a desolvation map), were centered on the binding site and were chosen to be large enough (60 × 60 × 60 Å) to allow the ligand to rotate freely, even in its most fully extended conformation. Docking was performed using the AutoDock empirical free energy function and the Lamarckian genetic algorithm with local search. Lamarckian genetic algorithms can handle ligands with more degrees of freedom than the simulated annealing method. In total, 150 docking runs with 2,500,000 energy evaluations for each run were performed for each molecule and all the evaluated HDACs. Cluster analysis (root-mean-square (RMS) tolerance equal to 0.5 Å) was then carried out on the docked results. Inhibitors were compared according to the cluster with lowest docked energy found. The inhibition constants (*K*i) were calculated from the docked energy.

The same protocol was used as in previous studies [49].

## 4. Conclusions

A series of novel compounds bearing an aza-heterocyclic moiety bound to the azelayl scaffold by an amide bond was synthesized to evaluate the biological effects induced on a panel of both normal and tumor cell lines. Noteworthy, none of the compounds induced cytotoxicity in the normal fibroblast cell line, while only osteosarcoma (U2OS) among the tumor cell lines appeared to be sensitive to compounds such as **5a**, **6**, and **8a**. The treatment with the compounds **5a** and **8a** induced an accumulation in the S phase, while compound **6** caused an increase in the G0/G1 fraction with concomitant decrease in the G2/M phase, and these changes in the cell cycle were associated with a post-transcriptional modification of both H2/H3 and H4 histones. The above cited three active molecules underwent an in silico study using histone deacetylases as the molecular target, revealing that they were able to interact with HDAC 7. These findings are in line with the recent studies, which disclosed an unexpected function for HDAC7 in osteoclasts, which is distinct from the function of HDAC3. Interestingly, compound **6** dramatically affected the FS signal of U2OS nuclei, indicating a change of the nuclei in both shape and size. Overall, compounds **5a**, **6**, and **8a** could be selective HDACi, achieving a greater clinical utility by elimination or reduction of serious side effects, associated with the current non-selective first-generation HDACi.

## Figures and Tables

**Table 1:** Half maximal inhibitory concentration (IC_50_) of compounds in different cell lines after 48 h of treatment (μM). (n.a. = non active; DMSO = dimethyl sulfoxide).

Compound	Solvent	U2OS IC_50_ (µM)	HDFa IC_50_ (µM)	HT29 IC_50_ (µM)	PC3 IC_50_ (µM)	IGROV1 IC_50_ (µM)
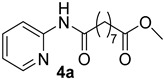	DMSO 60 mM	>100	n.a.	>100	n.a.	n.a.
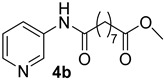	DMSO 60 mM	>100	n.a.	>100	n.a.	n.a.
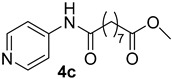	DMSO 60 mM	>100	n.a.	n.a.	n.a.	n.a.
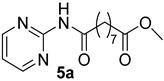	DMSO 60 mM	50	n.a.	n.a	n.a	n.a
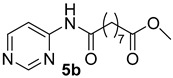	DMSO 60 mM	>100	n.a.	n.a.	n.a.	>100
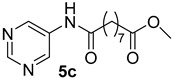	DMSO 60 mM	n.a.	n.a.	>100	>100	>100
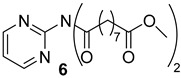	DMSO 60 mM	35	n.a.	>100	n.a.	n.a.
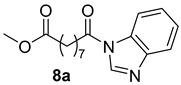	DMSO 60 mM	50	n.a.	>100	>100	n.a.
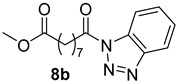	DMSO 60 mM	n.a.	n.a.	n.a.	n.a.	>100

**Table 2:** Docking results, expressed in term of binding free energy (∆G, kcal/mol) and calculated inhibition constant (*K*i, nM or μM).

	HDAC Class I				HDAC Class II	
	HDAC1-5ICN	HDAC2-4LXZ	HDAC3-4A69	HDAC8-4QA3	HDAC4-2VQM	HDAC7-3C0Z
BE_∆G (mol_**5a**)	−5.80	−6.90	−5.47	−8.05	−6.03	−7.26
∆G (mol_**6**)	−6.36	−7.24	−5.92	−8.14	−7.61	−7.00
∆G (mol_**8a**)	−6.83	−7.73	−6.38	−8.63	−6.70	−7.61
*K*i (mol_**5a**)	55.93 μM	8.77 μM	98.23 μM	1.26 μM	37.85 μM	4.78 μM
*K*i (mol_**6**)	21.78 μM	4.94 μM	45.52 μM	1.07 μM	2.62 μM	7.37 μM
*K*i (mol_**8a**)	9.92 μM	2.17 μM	21.08 μM	472.15 nM	12.29 μM	2.62 μM

BE_∆G: binding energy (kcal/mol); *K*i: calculated inhibition constant.

## Supplementary Materials

The following are available online: Figures S1–S27: ^1^H- and ^13^C-NMR spectra of compounds **4a**–**c**, **5a**–**c**, **6**, **8a**, **8b**; Figure S28: docking pose for **8a** in HDAC2; Figure S29: Docking pose for molecule **5a** in binding site of HDAC8, HADC7, and HDAC2; Figure S30: Docking pose for molecule **6** in binding site of HDAC8, HADC7, and HDAC2; Figure S31: Docking pose for all the evaluated compounds in the HDAC8 and HDAC7 active binding sites.
