# Supplementary material for: Synthesis of Novel Structural Hybrids between Aza-Heterocycles and Azelaic Acid Moiety with a Specific Activity on Osteosarcoma Cells
Source: Molecules. 2020 Jan 18;25(2):404. doi: 10.3390/molecules25020404 (PMC7024557; doi:10.3390/molecules25020404)
Supplement: Supplementary file 1 [file molecules-25-00404-s001.pdf]

Synthesis of novel structural hybrids between aza-heterocycles and azelaic acid moiety with a specific activity on osteosarcoma cells

Gabriele Micheletti[1]*, Natalia Calonghi[2]*, Giovanna Farruggia[2,3], Elena Strocchi[1], Vincenzo Palmacci[1], Dario Telese[1], Silvia Bordoni[1], Giulia Frisco[2], Carla Boga[1]

[1] Department of Industrial Chemistry 'Toso Montanari', Alma Mater Studiorum Università di Bologna Viale Del Risorgimento, 4 402136 Bologna Italy; (GM), carla.boga@unibo.it (CB), dario.telese2@unibo.it (DT), silvia.bordoni@unibo.it (SB), vincenzo.palmacci@studio.unibo.it; elena.strocchi@unibo.it (ES)

[2] Department of Pharmacy and Biotechnology, University of Bologna, Via Irnerio 48,40126 Bologna (Italy); (NC), giovanna.farruggia@unibo.it (GF1), giulia.frisco2@unibo.it (GF2)

[3] National Institute of Biostructures and Biosystems, Viale delle Medaglie d'Oro, 305, Rome 00136, Italy (GF)

Supporting information

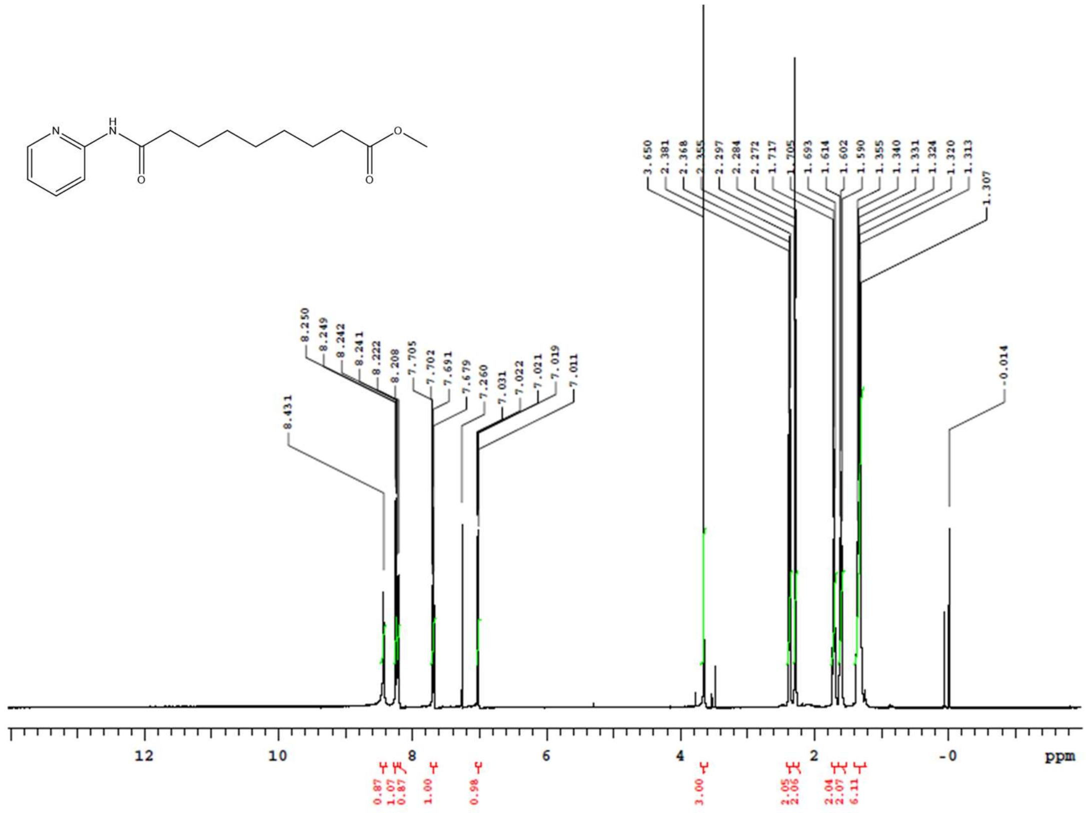

Figure S1. ^{1}HNMR spectrum ($CDCl_3$, 600 MHz) of compound **4a**

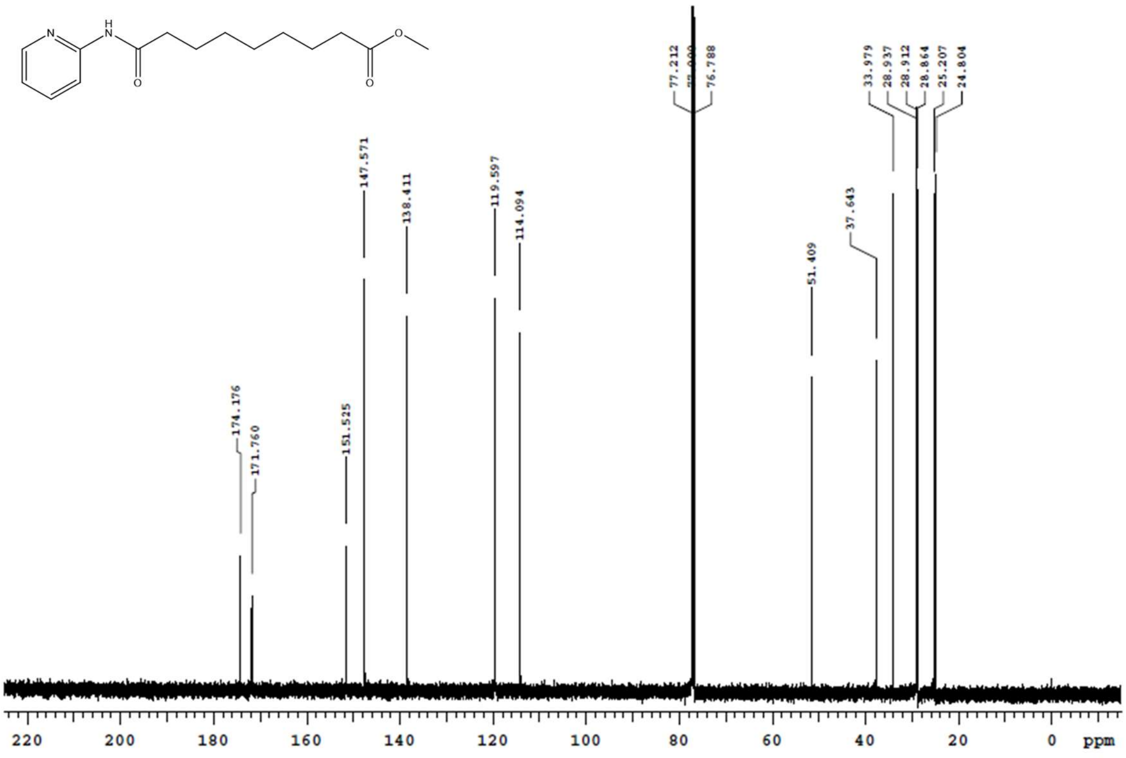

Figure S2. ^{13}CNMR spectrum ($CDCl_3$, 150 MHz) of compound **4a**

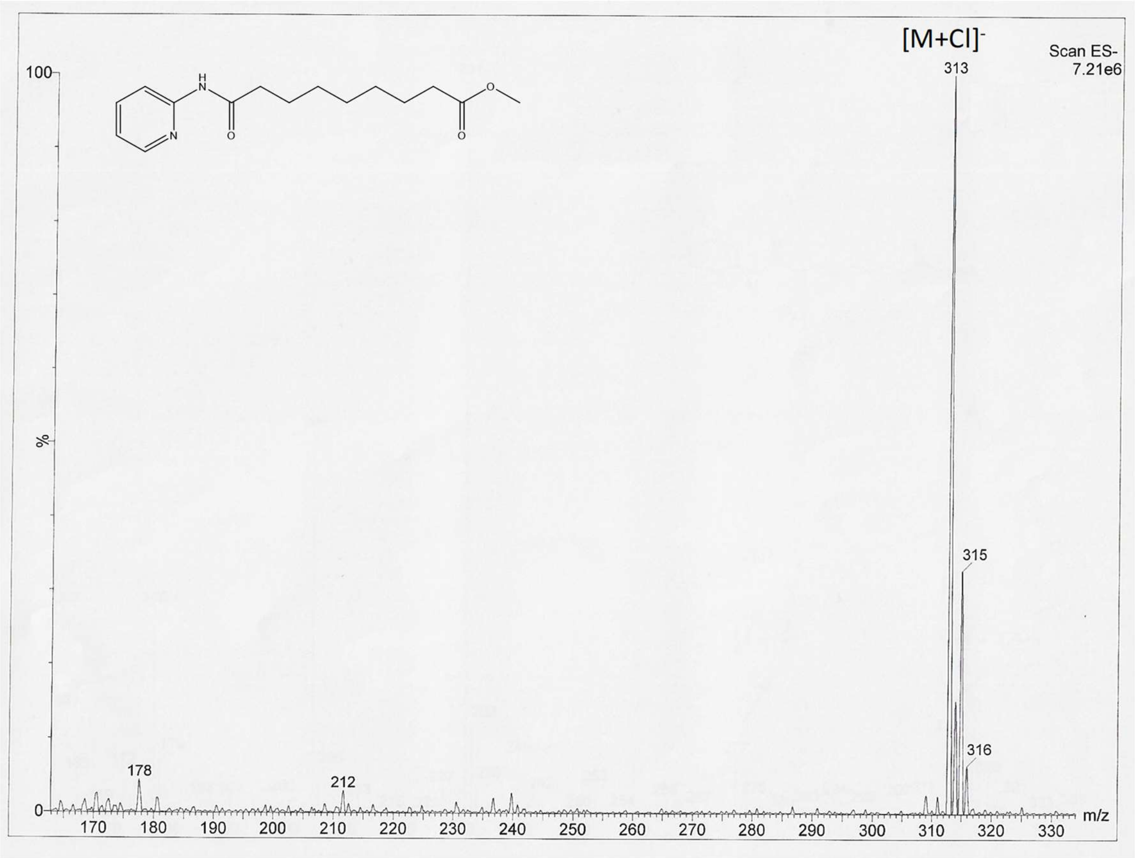

Figure S3. ESI-MS- spectrum of compound **4a**

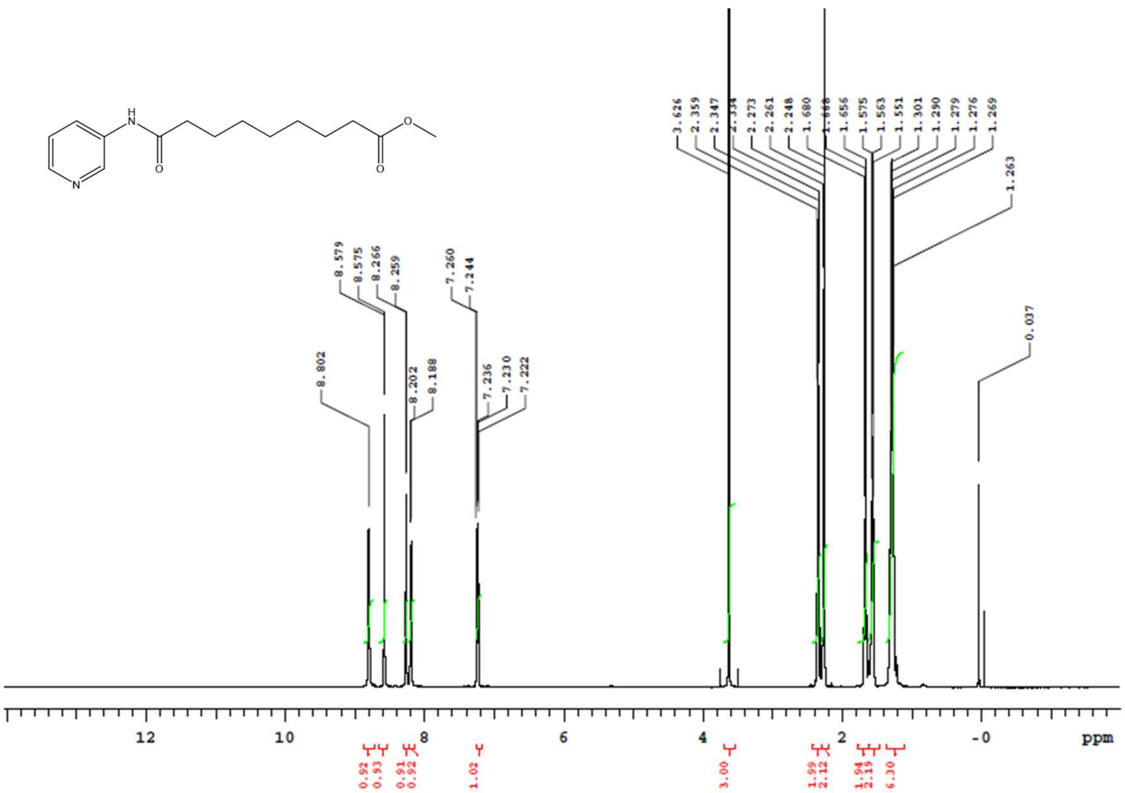

Figure S4. ^{1}HNMR spectrum ($CDCl_3$, 600 MHz) of compound **4b**

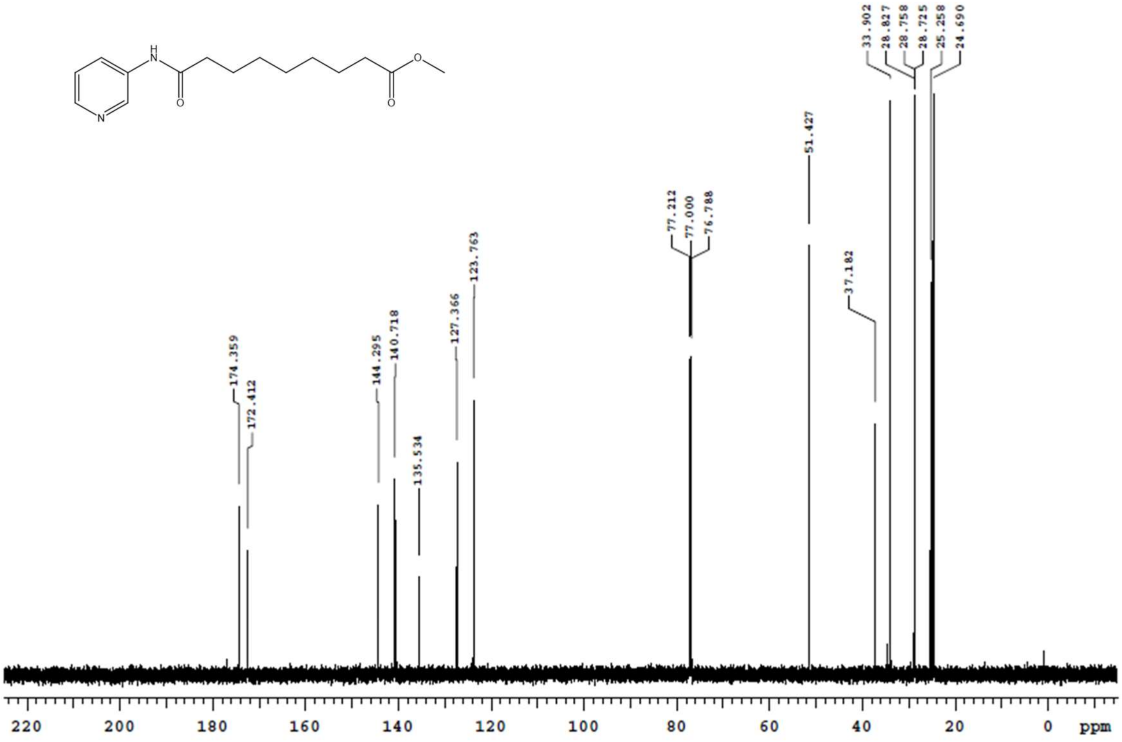

Figure S5. ^{13}CNMR spectrum ($CDCl_3$, 150 MHz) of compound **4b**

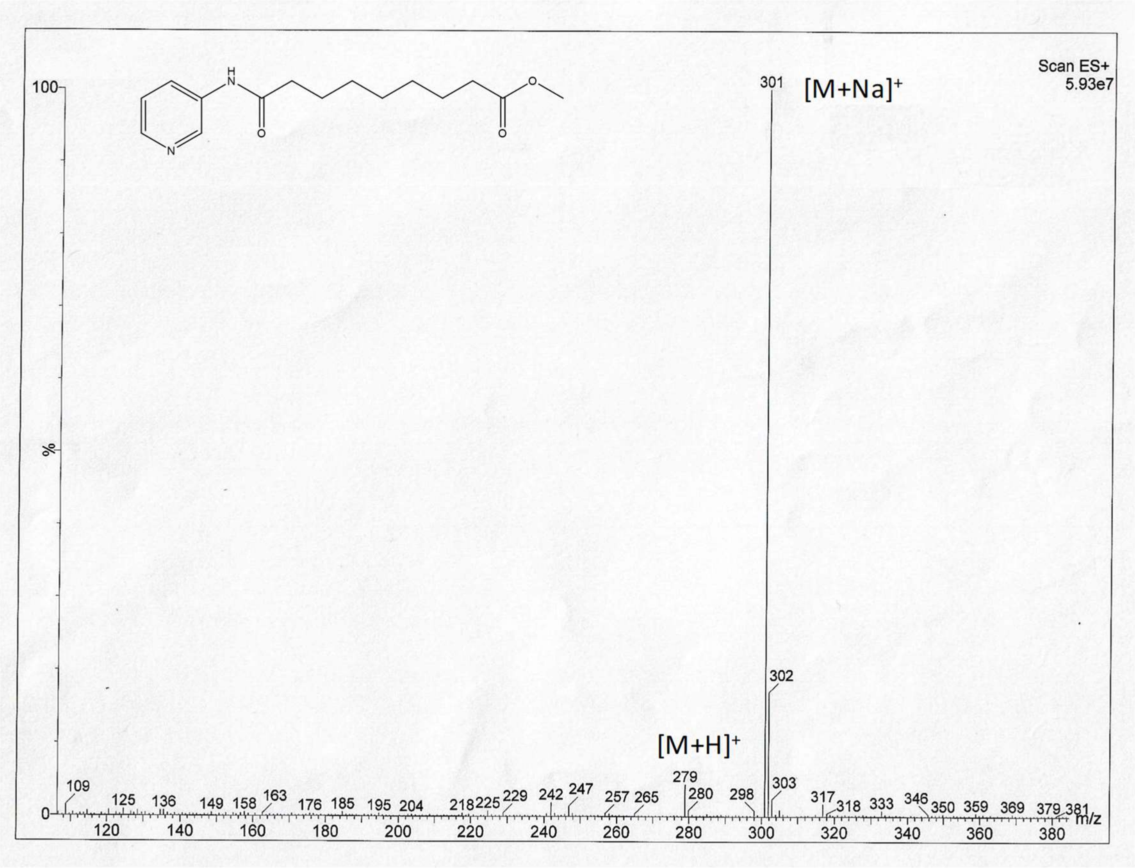

Figure S6. ESI-MS+ spectrum of compound **4b**

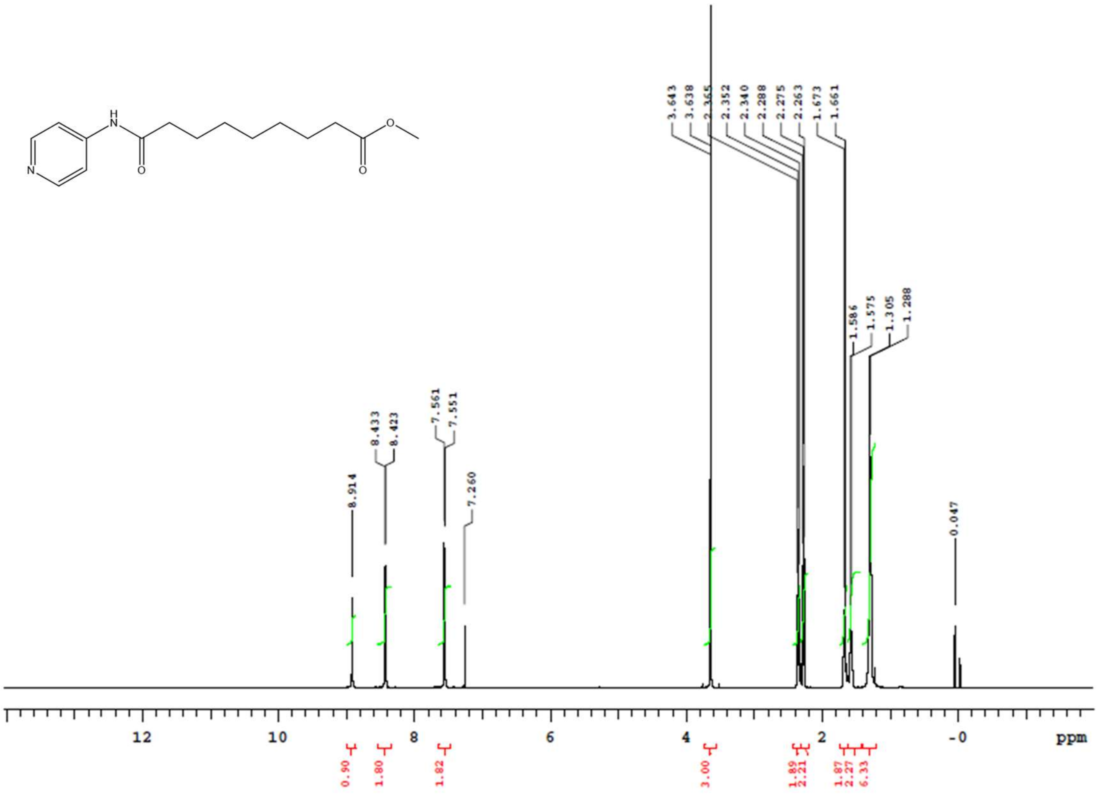

Figure S7. ^{1}HNMR spectrum ($CDCl_3$, 600 MHz) of compound **4c**

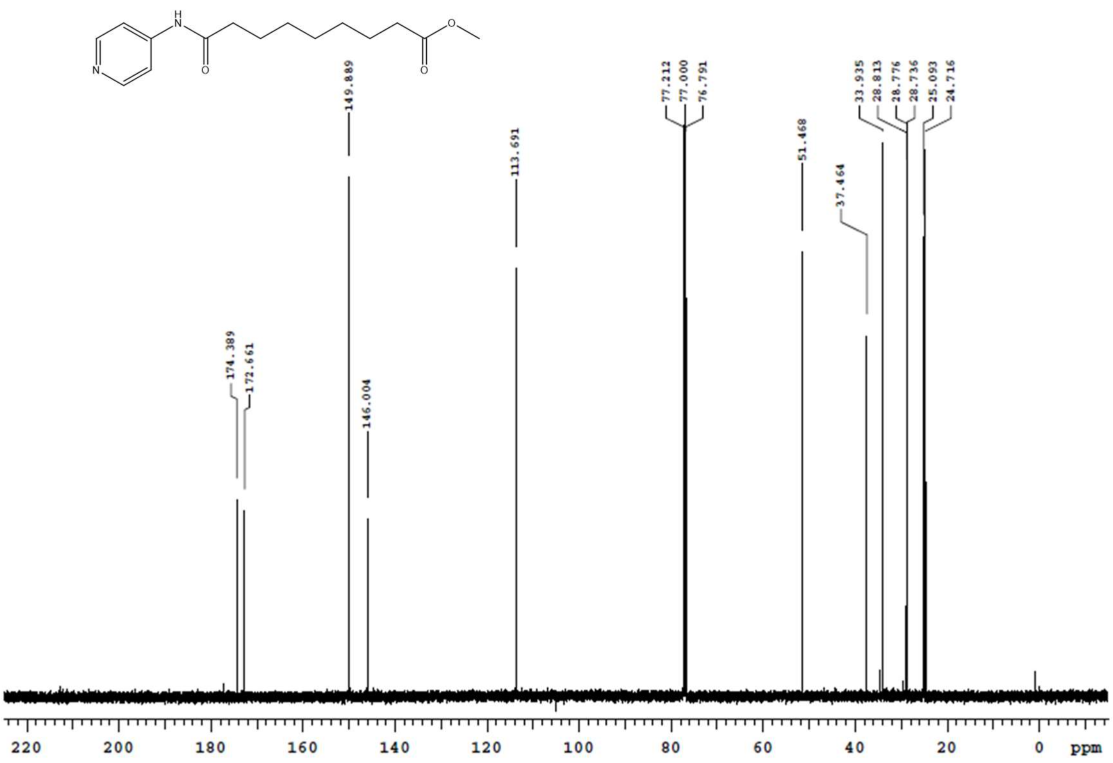

Figure S8. ^{13}CNMR spectrum ($CDCl_3$, 150 MHz) of compound **4c**

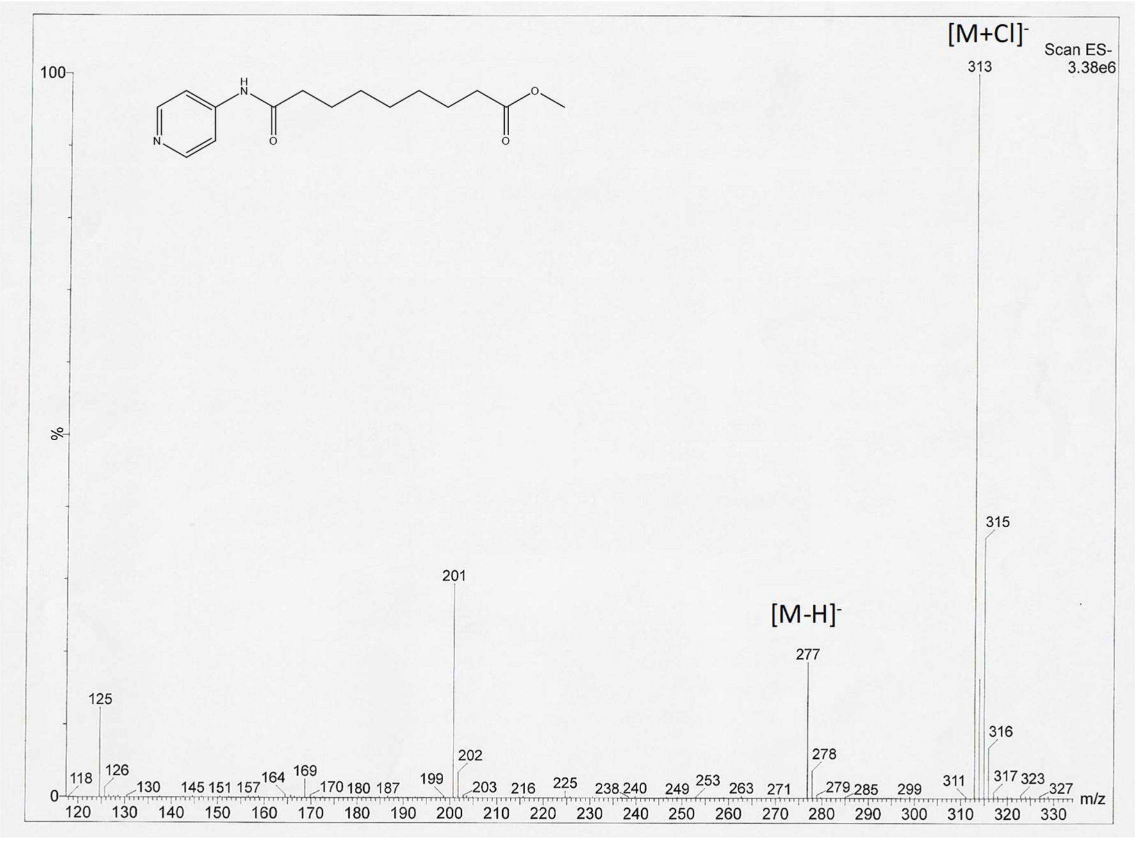

Figure S9. ESI-MS^{-} spectrum of compound **4c**

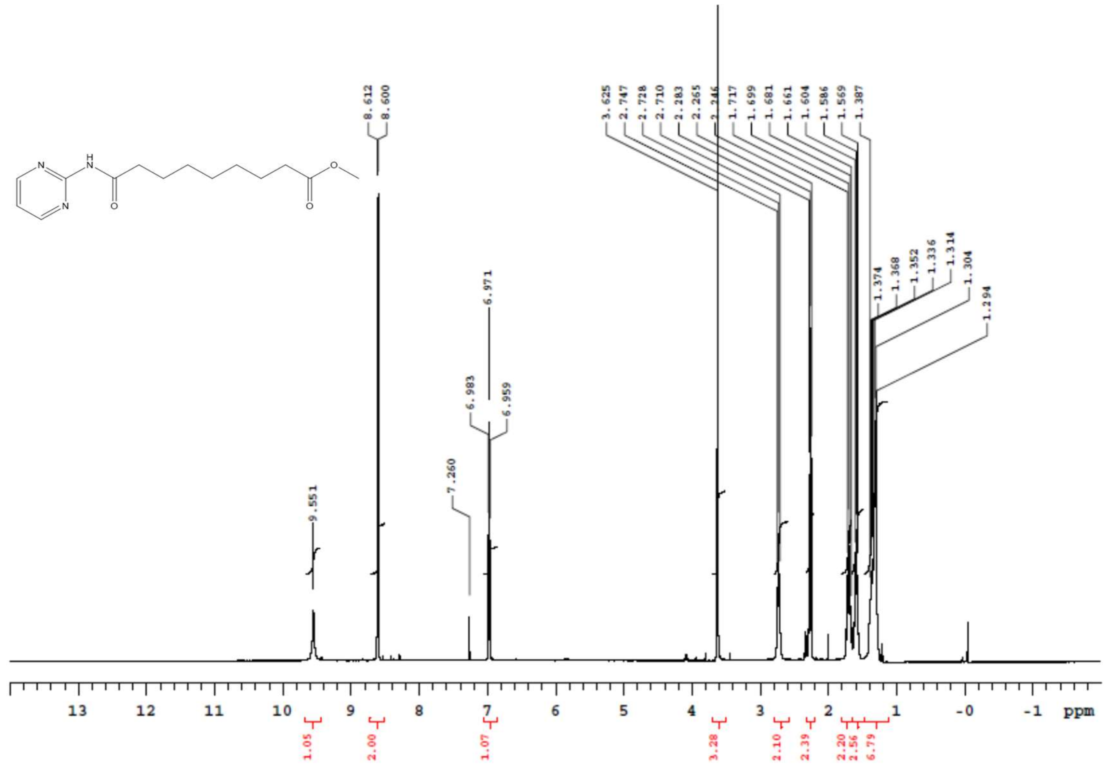

Figure S10. ^{1}HNMR spectrum ($CDCl_3$, 400 MHz) of compound **5a**

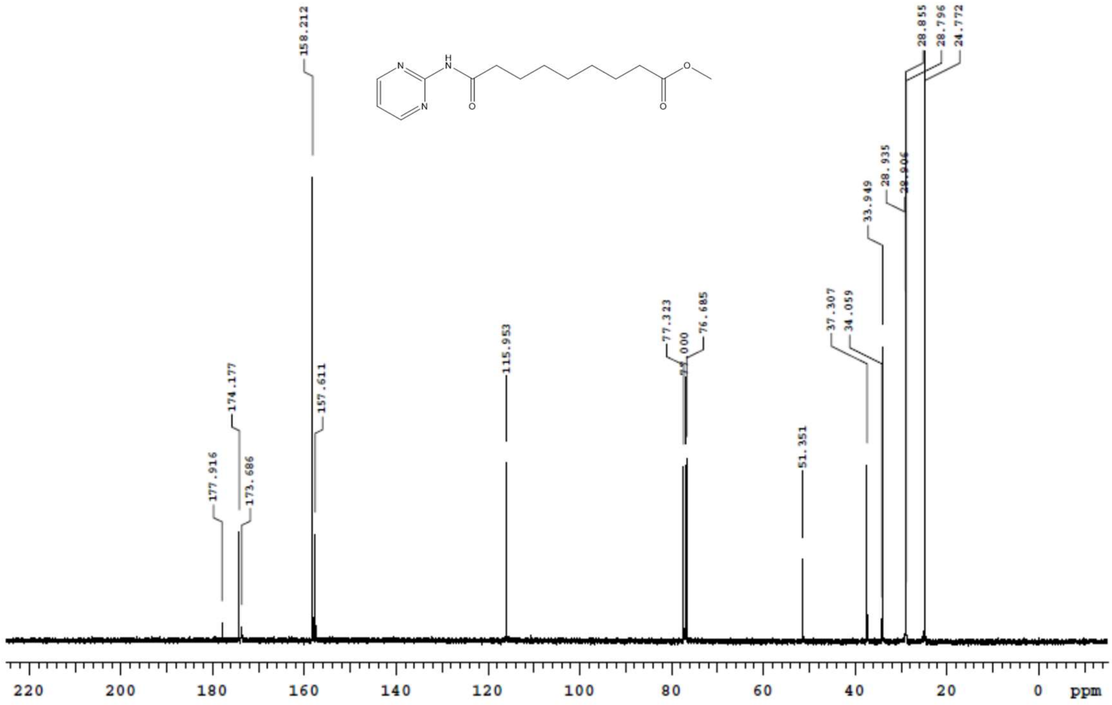

Figure S11. ^{13}CNMR spectrum ($CDCl_3$, 100 MHZ) of compound **5a**

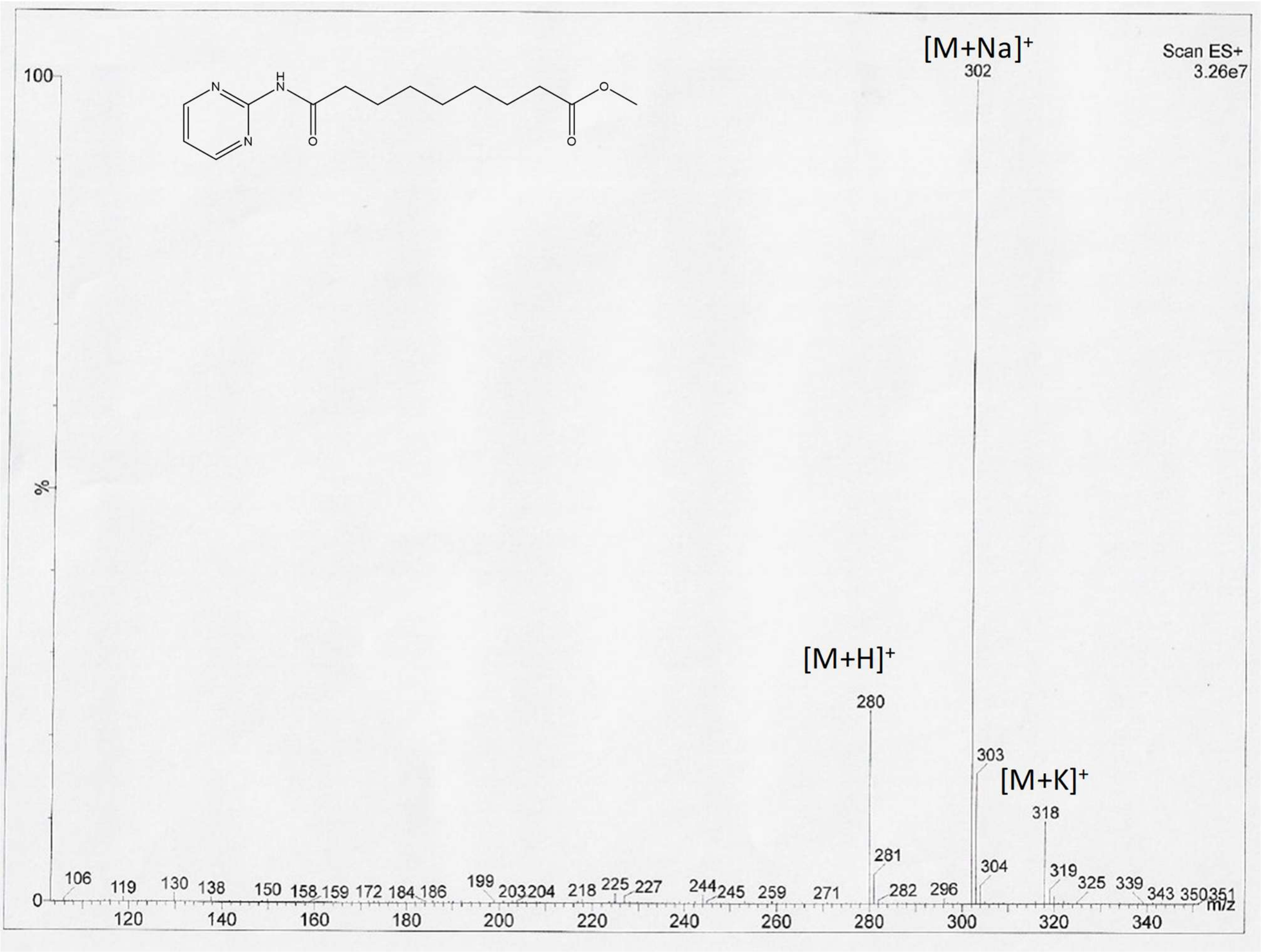

Figure S12. ESI-MS+ spectrum of compound **5a**

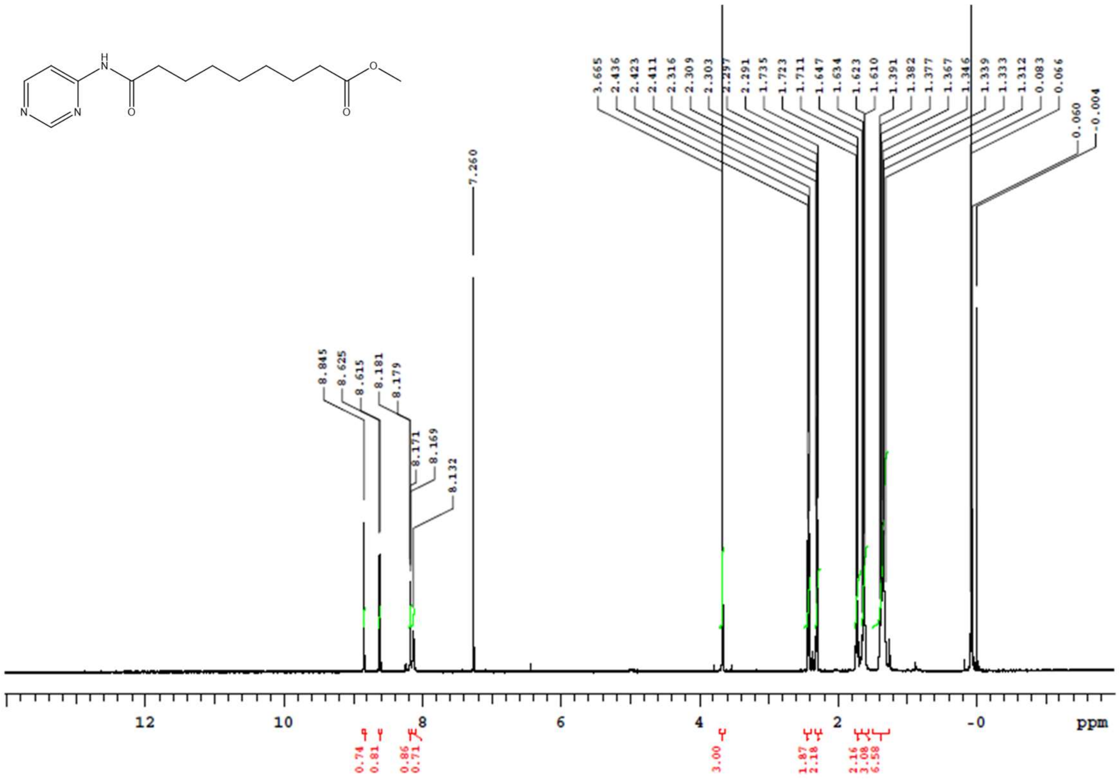

Figure S13. ^{1}HNMR spectrum ($CDCl_3$, 600 MHz) of compound **5b**

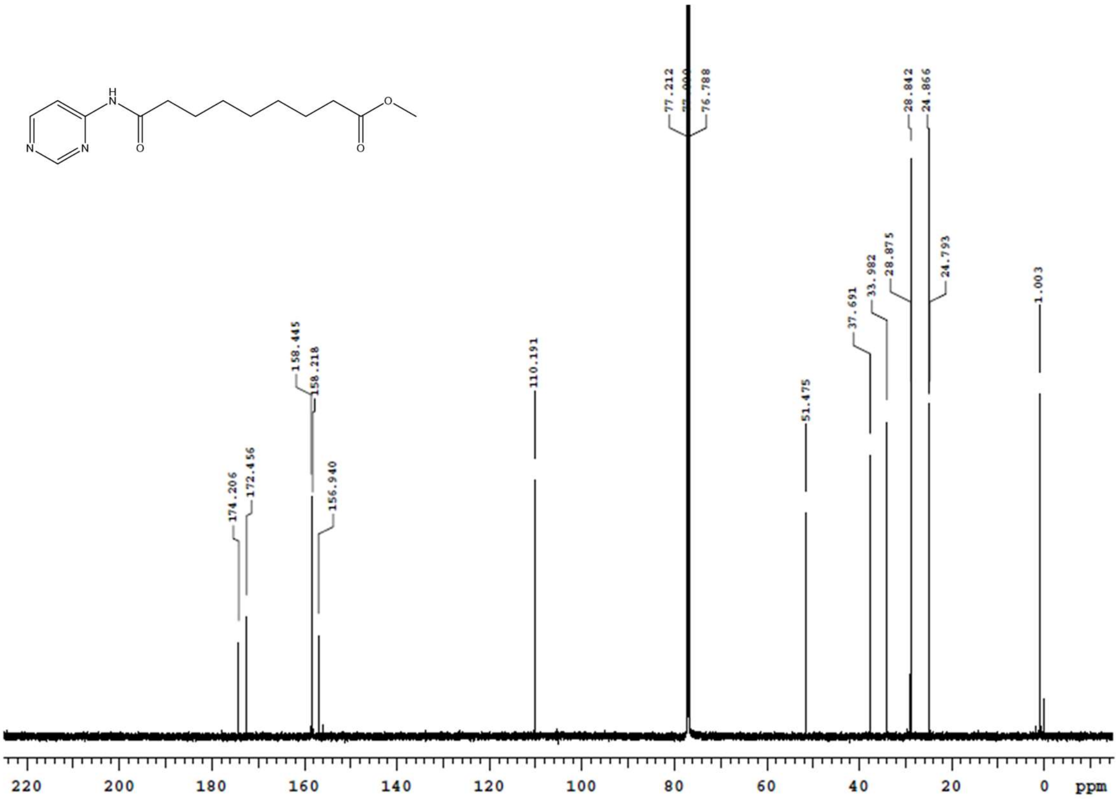

Figure S14. ^{13}CNMR spectrum ($CDCl_3$, 150 MHz) of compound **5b**

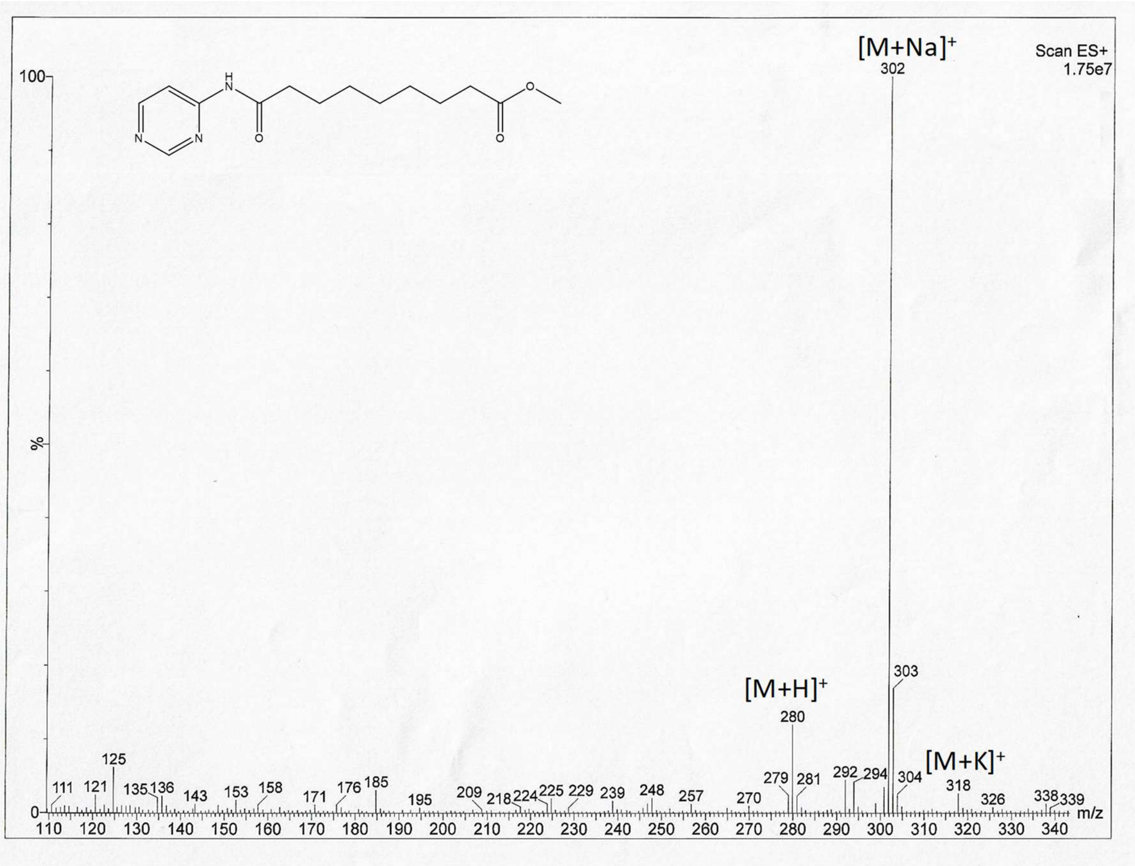

Figure S15. ESI-MS$^+$ spectrum of compound **5b**

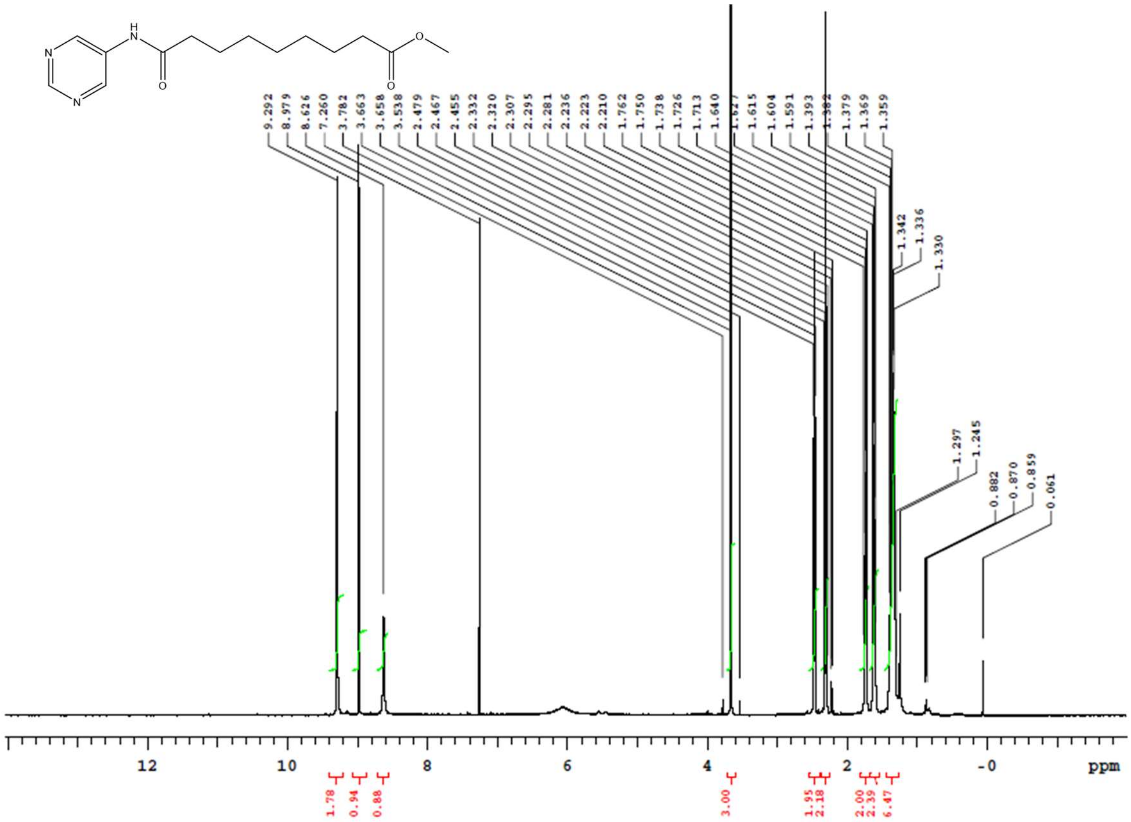

Figure S16. ^{1}HNMR spectrum ($CDCl_3$, 600 MHz) of compound **5c**

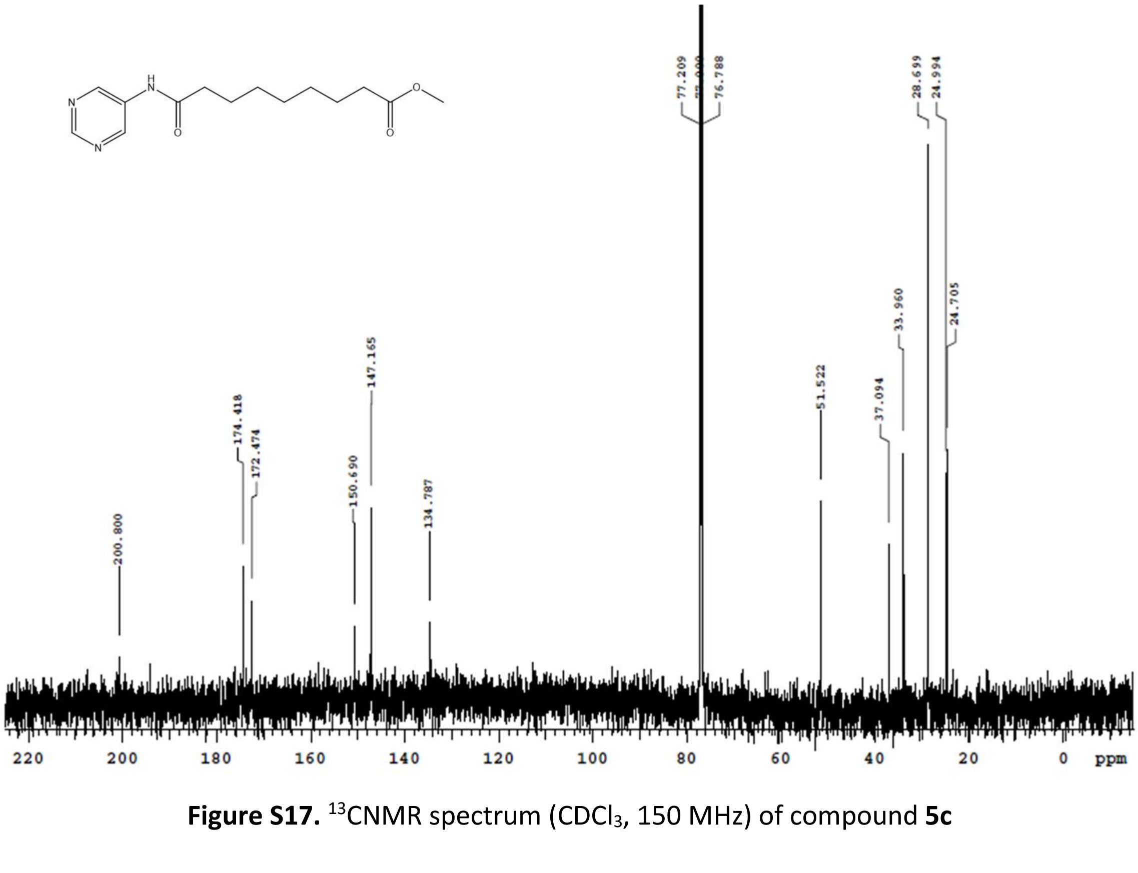

Figure S17. ^{13}CNMR spectrum ($CDCl_3$, 150 MHz) of compound **5c**

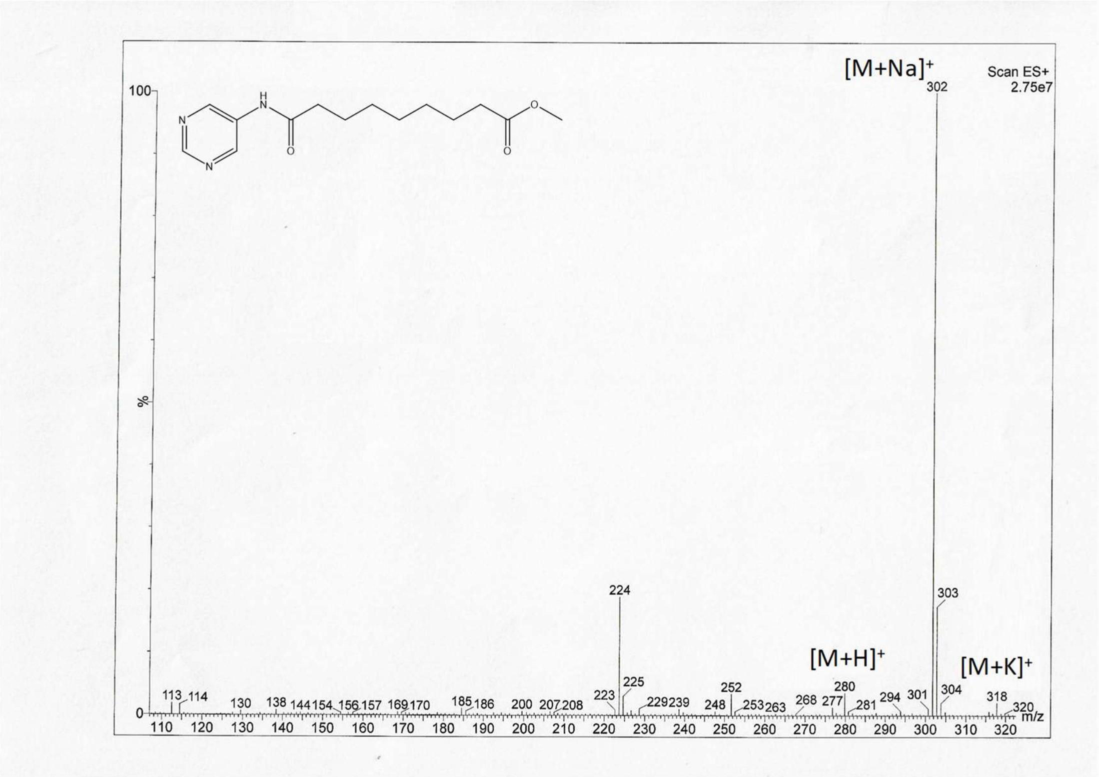

Figure S18. ESI-MS+ spectrum of compound **5c**

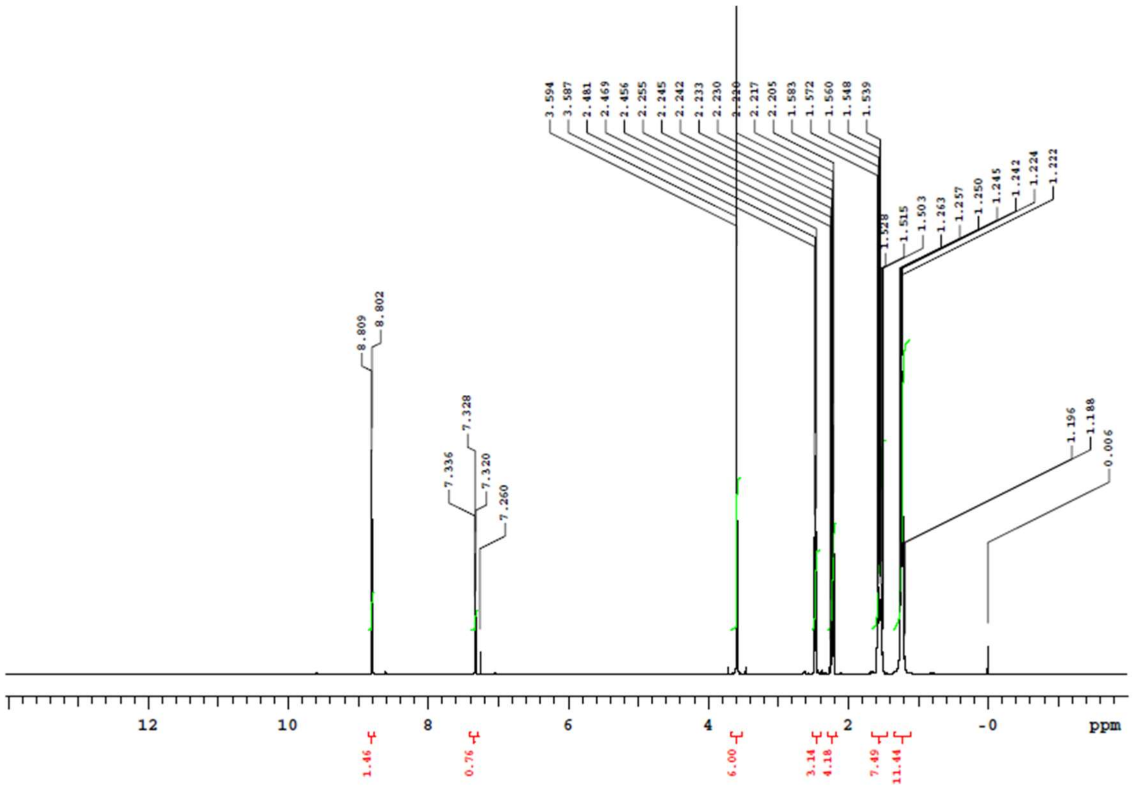

Figure S19. ^{1}HNMR spectrum ($CDCl_3$, 600 MHz) of compound **6**

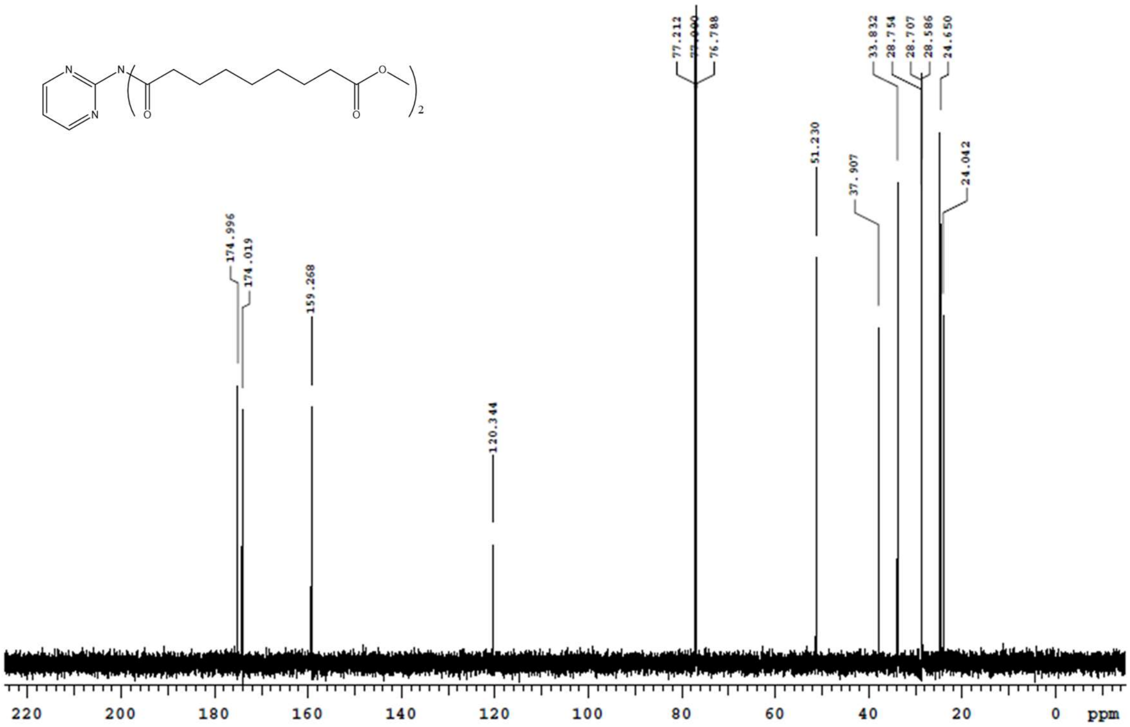

Figure S20. ^{13}CNMR spectrum ($CDCl_3$, 150 MHZ) of compound **6**

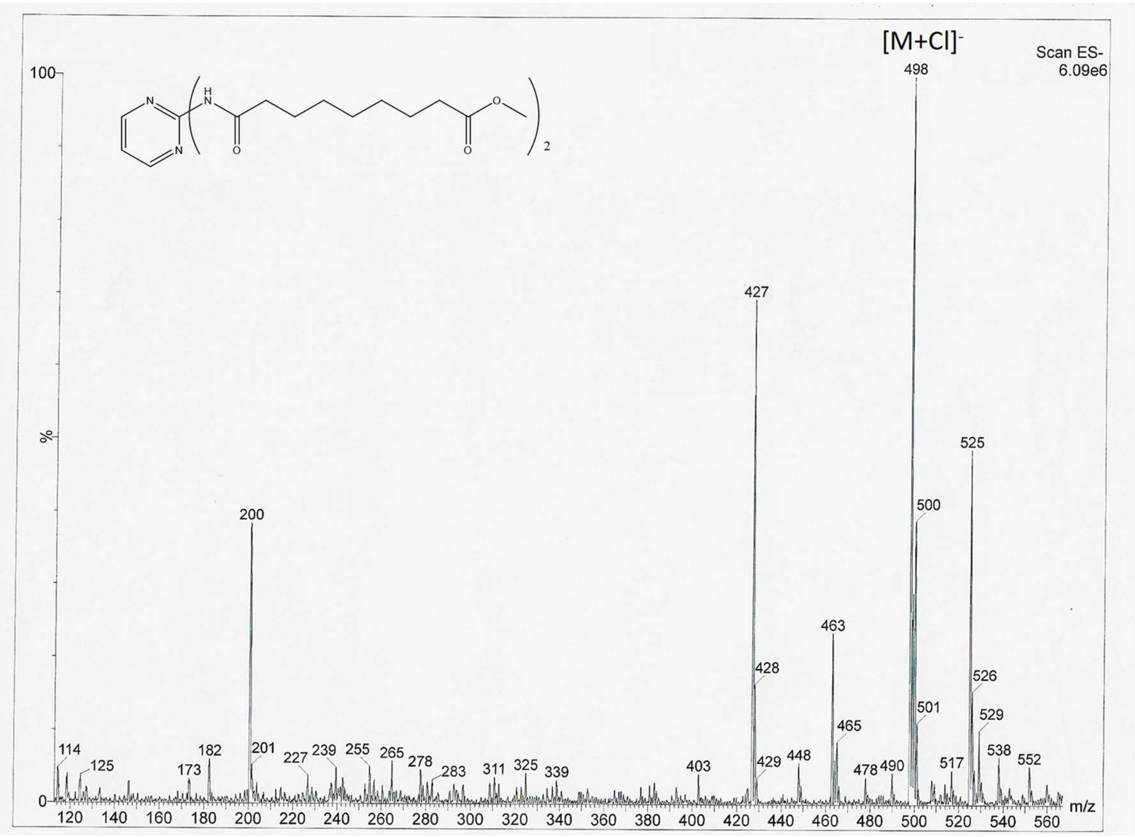

Figure S21. ESI-MS$^-$ spectrum of compound **6**

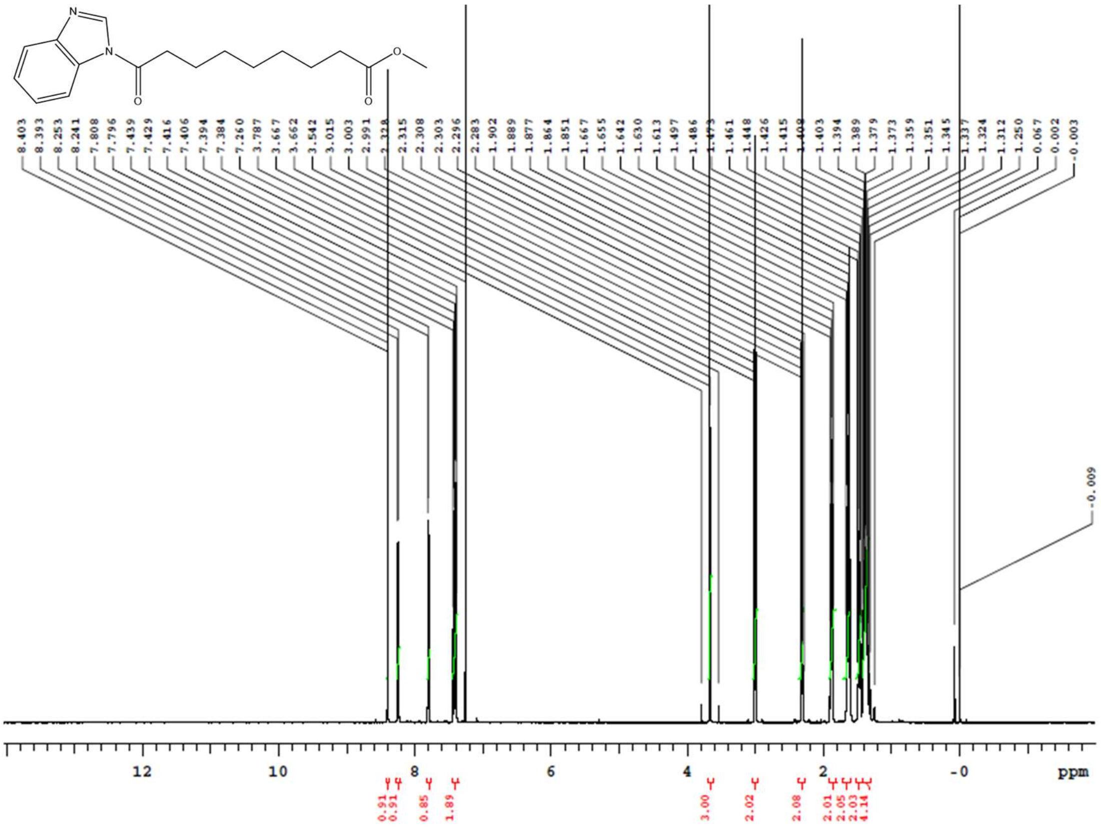

Figure S22. ^{1}HNMR spectrum ($CDCl_3$, 600 MHz) of compound **8a**

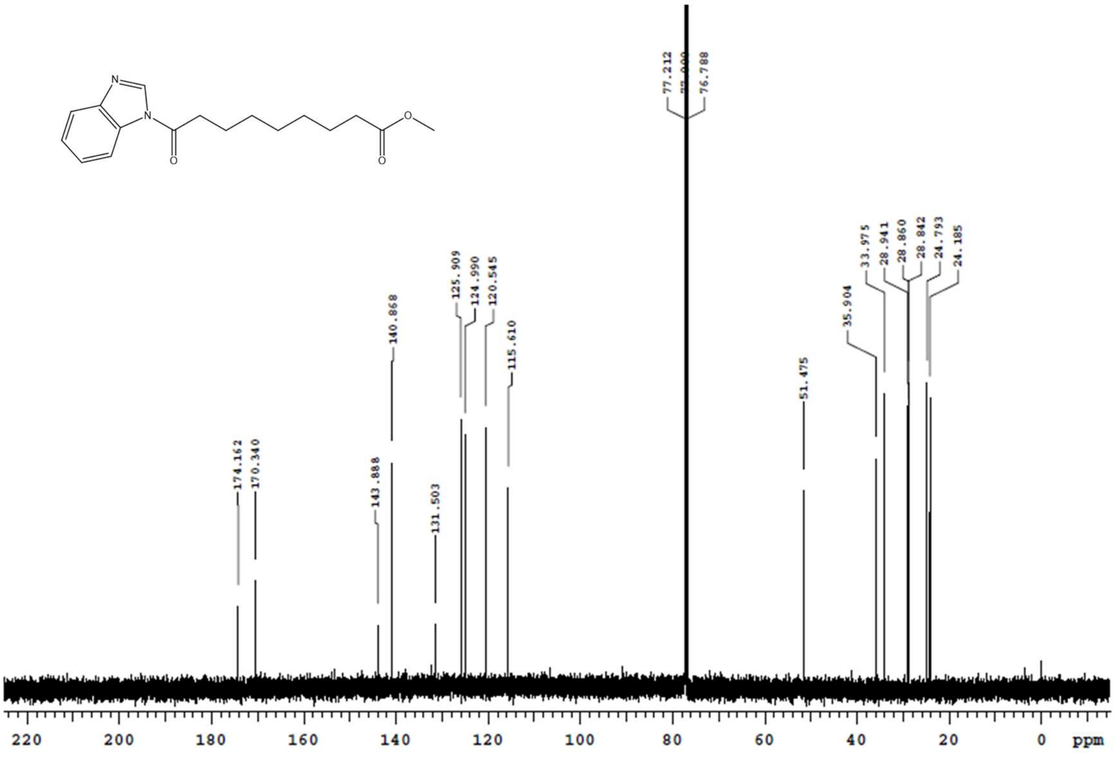

Figure S23. ^{13}CNMR spectrum ($CDCl_3$, 150 MHz) of compound **8a**

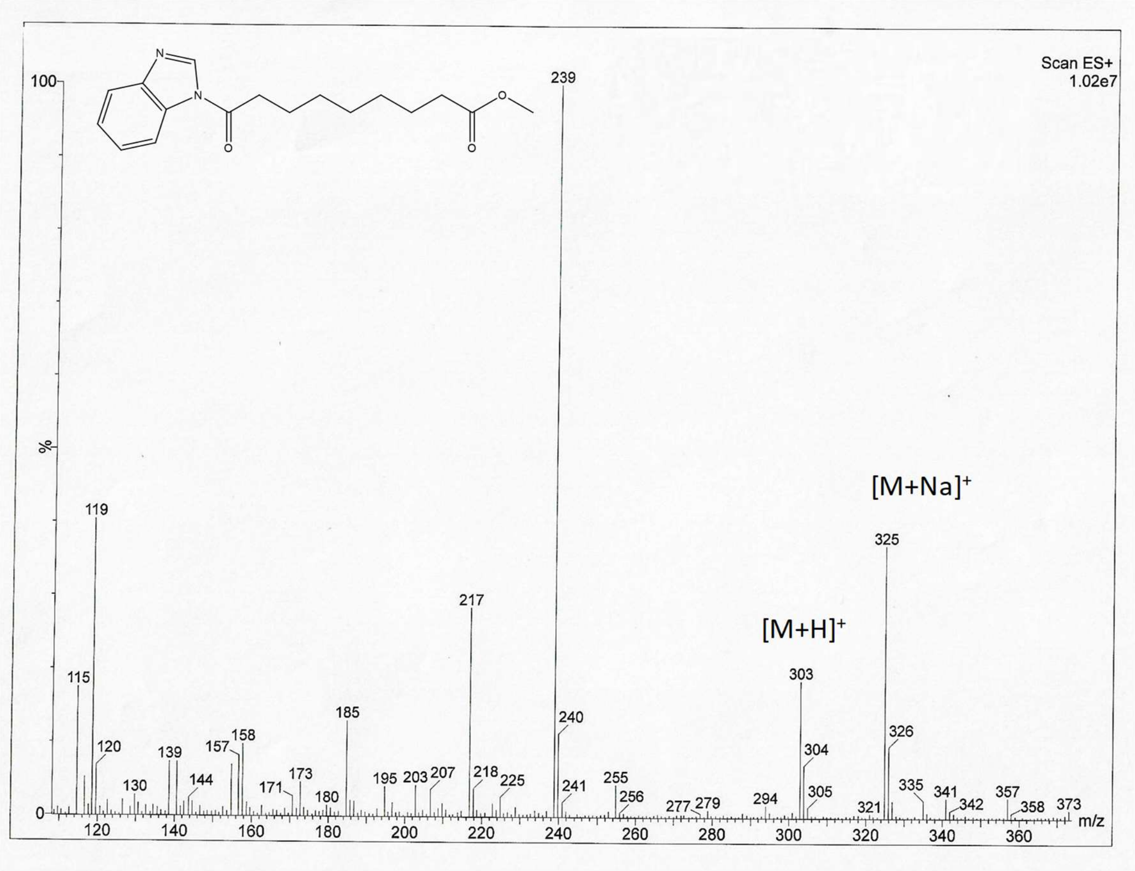

Figure S24. ESI-MS+ spectrum of compound **8a**

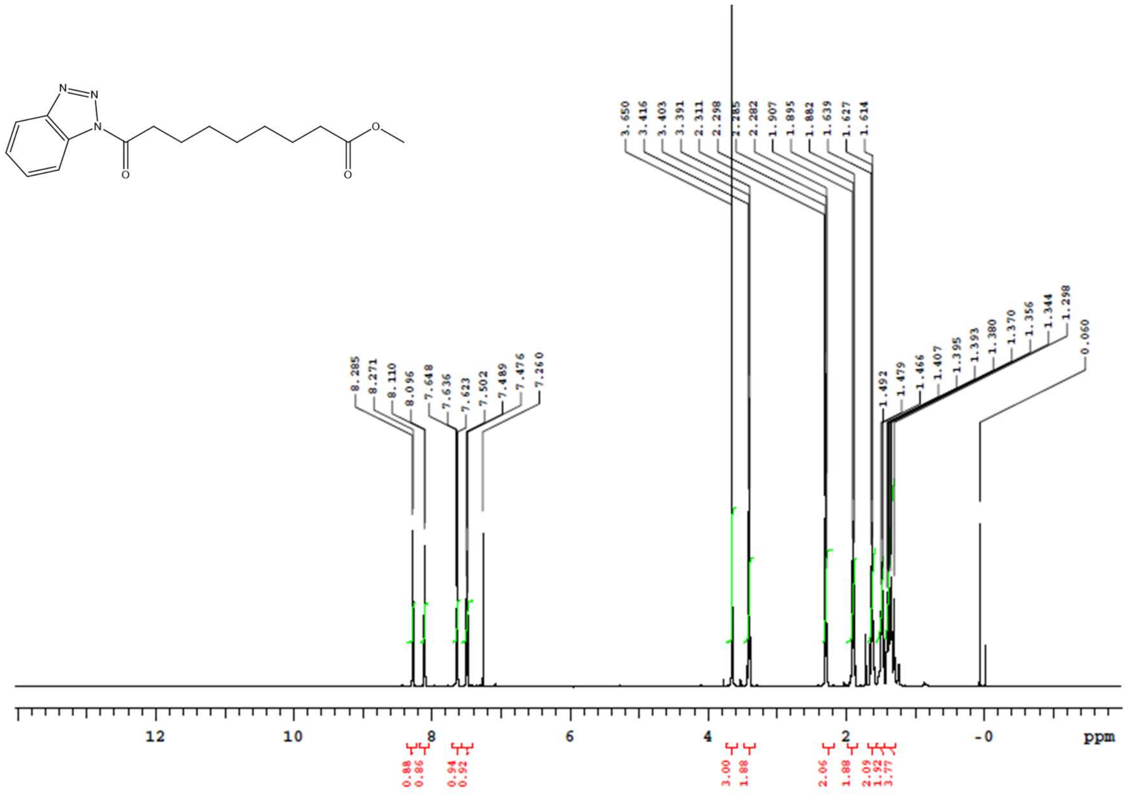

Figure S25. ^{1}HNMR spectrum ($CDCl_3$, 600 MHz) of compound **8b**

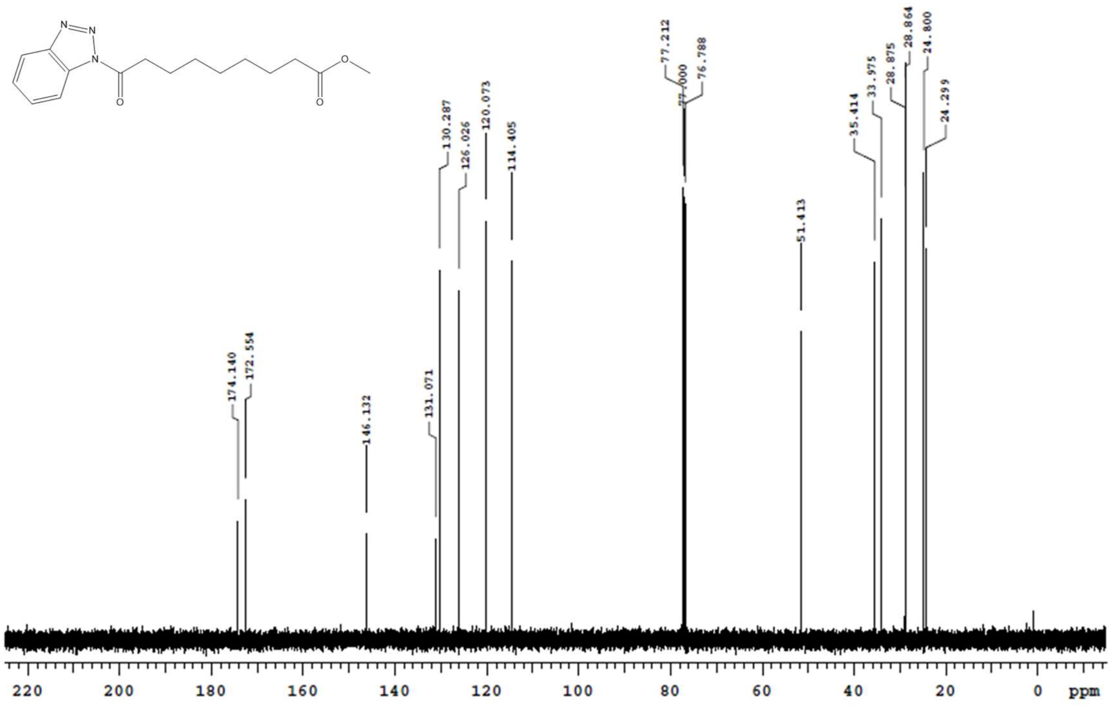

Figure S26. ^{13}CNMR spectrum ($CDCl_3$, 150 MHZ) of compound **8b**

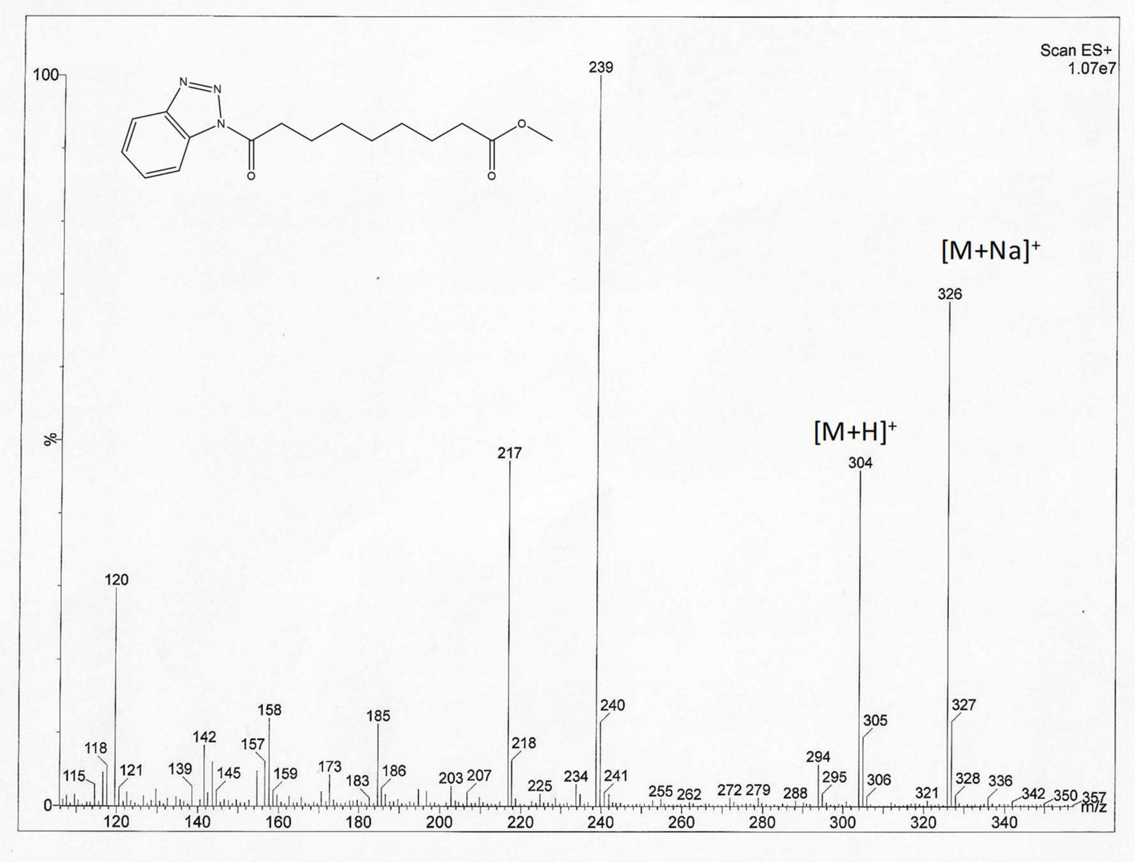

Figure S27. ESI-MS$^+$ spectrum of compound **8b**

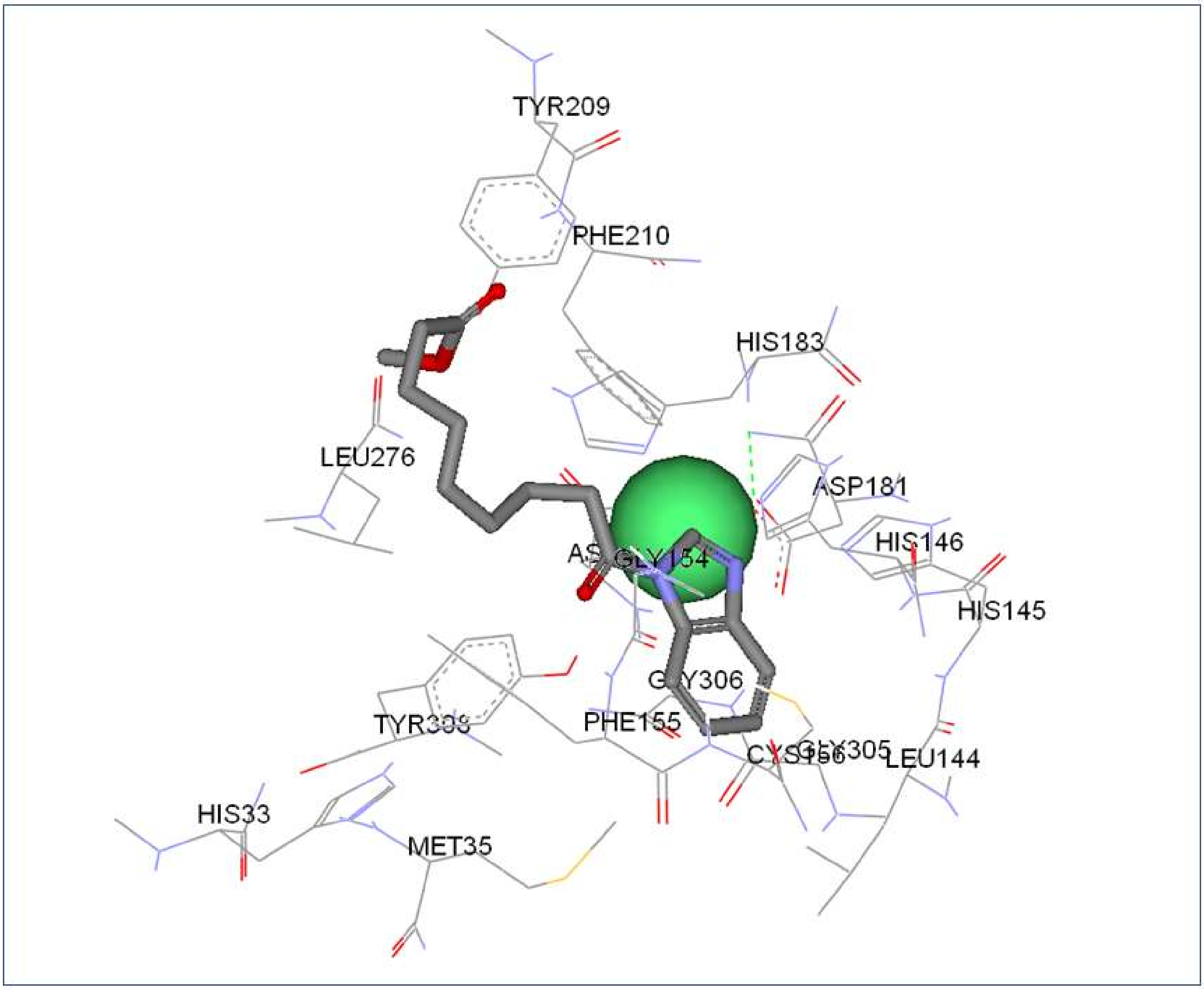

Figure S28. Docking poses of compound **8a** into the binding site of HDAC2 structure (PDB ID: 4LXZ)

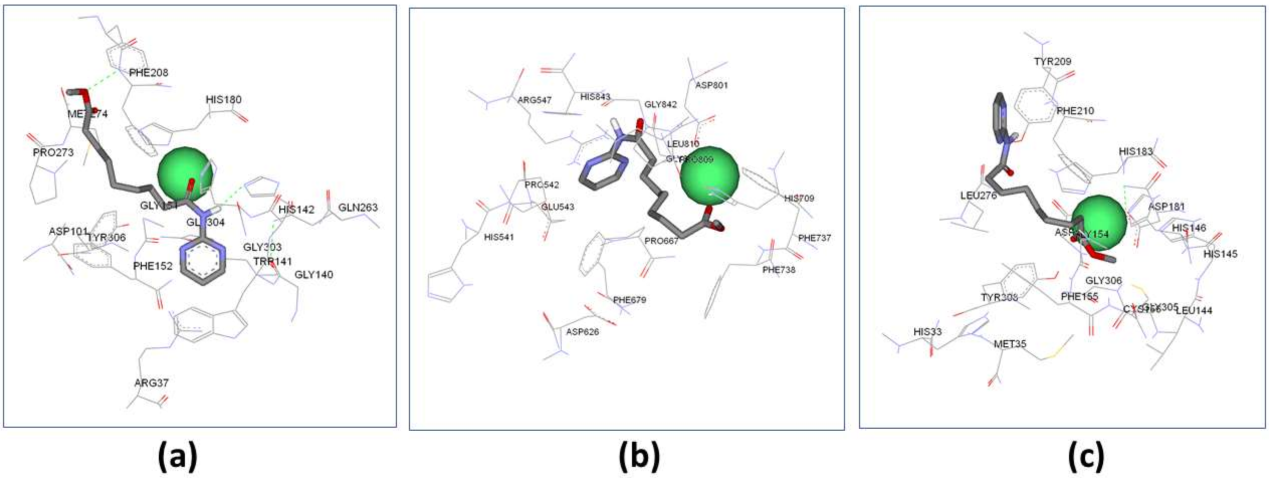

Figure S29. Docking poses of compound **5a** into the binding site of: (**a**) HDAC8 structure (PDB ID: 4QA3); (**b**) HDAC7 structure (PDB ID: 3C0Z) and (**c**): HDAC2 structure (PDB ID: 4LXZ), respectively. In both pictures zinc ion is represented as green sphere.

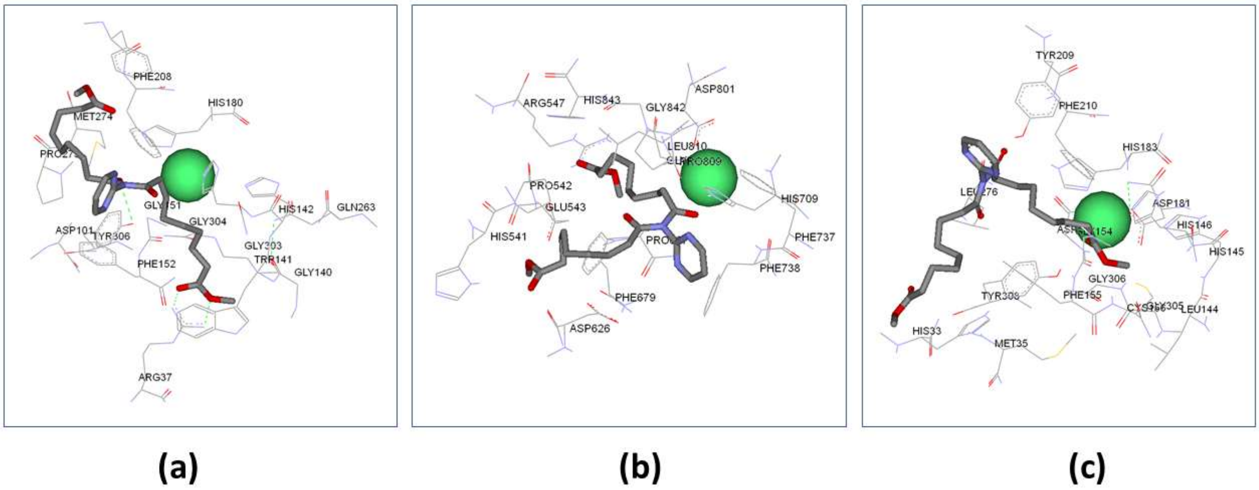

Figure S30. Docking poses of compound **6** into the binding site of: (**a**) HDAC8 structure (PDB ID: 4QA3); **(b)** HDAC7 structure (PDB ID: 3C0Z) and (**c**): HDAC2 structure (PDB ID: 4LXZ), respectively. In both pictures zinc ion is represented as green sphere**.**

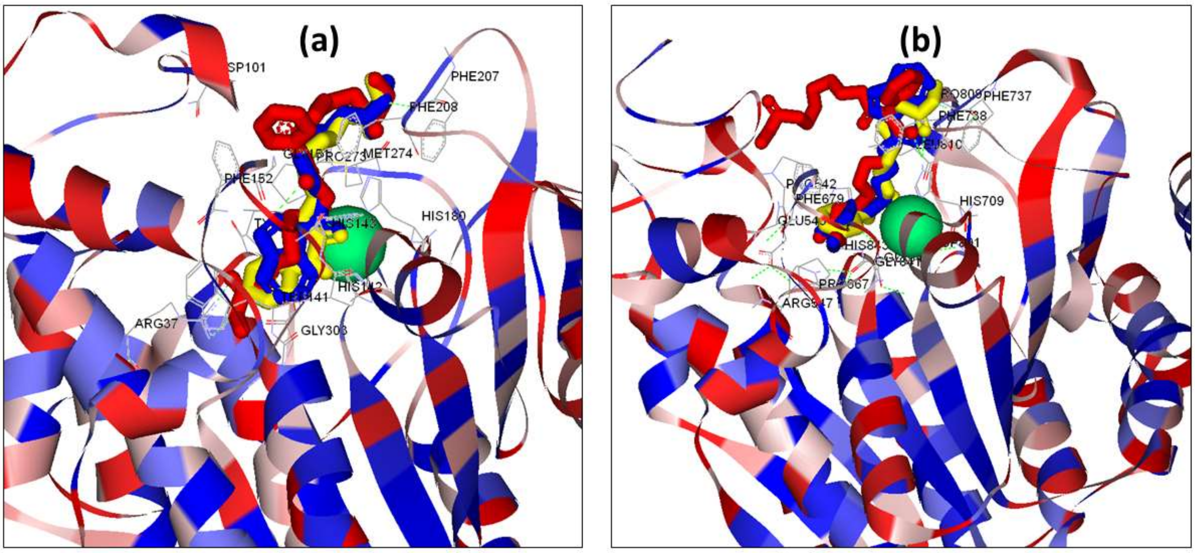

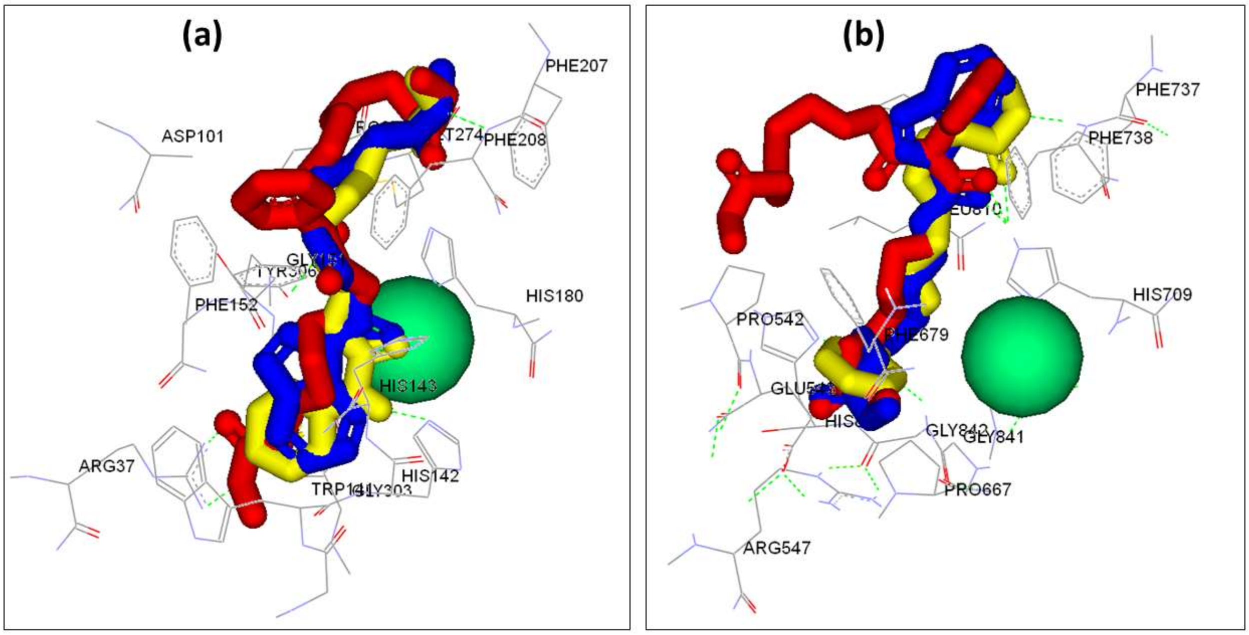

Figure S31: docking pose for all the evaluated compounds into the HDAC8 and HDAC7 active binding sites in two different representations modes (top and bottom). (**a**): Docking poses of compounds **5a** (yellow), **6** (red) and **8a** (blue) in the binding site of HDAC8 structure (PDB ID: 4QA3). Hydrogen bonds and zinc ion are represented as green dash line and green sphere, respectively. (**b**): Docking poses of compounds **5a** (yellow), **6** (red) and **8a** (blue) in the binding site of HDAC7 structure (PDB ID: 3C0Z). Hydrogen bonds and zinc ion are represented as green dash line and green sphere, respectively.

Table S1. Hydrogen bonds and virtual interactions within 5 Å between compound **5a**, **6**, and **8a** and aminoacid residues (and zinc ion) into the binding site of HDAC8 structure (PDB ID: 4QA3) obtained from docking evaluation.

**HDAC8-4QA3 aminoacidic residues in the active site**	**5a**	**6**	**8a**
ARG37 (positively charged) $(-(CH_2)_3-NH-C(-NH_2)=NH_2^+$		near to $-COOCH_3$ group belonging to chain a	
ASP101 negatively charged $(-COO^-)$		near to pyridimidinyl ring	
GLY140	near to pyrimidinyl ring		near to benzimidazolyl moiety
TRP141	near to pyrimidinyl ring	near to $-COOCH_3$ group belonging to chain a	near to benzimidazolyl moiety
HIS142	near to oxygen atom of amide group		near to benzimidazolyl moiety
HIS143	near to oxygen atom of amide group		near to benzimidazolyl moiety
GLY151		near to amide C=O group belonging to chain a	
PHE152		near to pyridimidinyl ring	

**HIS180**	**H bond with amidic C=O**	**H bond with amidic C=O**	**H bond with amidic C=O**
PHE207	near to $COOCH_3$ group	near to $COOCH_3$ group belonging to chain b	near to $COOCH_3$ group
PHE208	near to $COOCH_3$ group	near to pyridimidinyl ring	near to $COOCH_3$ group
PRO273	near to $COOCH_3$ group	near to $COOCH_3$ group belonging to chain b	near to $COOCH_3$ group
MET274		near to $COOCH_3$ group belonging to chain b	
GLY303	near to pyrimidinyl ring	near to $COOCH_3$ group belonging to chain a	near to benzimidazolyl moiety
GLY304	near to pyrimidinyl ring	near to $COOCH_3$ group belonging to chain a	near to benzimidazolyl moiety
**TYR306**	near to amide carbonyl	**H bond with amidic C=O**	near to benzimidazolyl moiety
Zn 502	near to oxygen atom of amide group	near to oxygen atom of amide group	near to oxygen atom of amide group

Table S2. Hydrogen bonds and virtual interactions within 5 Å between compound **5a**, **6**, and **8a** and aminoacid residues (and zinc ion) into the binding site of HDAC7 structure (PDB ID: 3C0Z) obtained from docking evaluation.

**HDAC7-3C0Z aminoacid residues in the active site**	**5a**	**6**[a]	**8a**
PRO542	near to pyrimidinyl ring	near to $COOCH_3$ group belonging to chain a	
GLU543 negatively charged ($-CH_2COO^-$)	near to pyrimidinyl ring	near to $COOCH_3$ group belonging to chain a	near to $COOCH_3$ group
ARG547 (positively charged) ($-(CH_2)_3-NH-C(-NH_2)=NH_2^+$		near to $COOCH_3$ group belonging to chain a	near to $COOCH_3$ group
PRO667			near to $COOCH_3$ group
PHE679	near to aliphatic chain	near to aliphatic chain a	near to aliphatic chain
**HIS709**	**H bond with amidic C=O**	**H bond with amidic C=O belonging to chain a**	**H bond with amidic C=O**
PHE737	near to $COOCH_3$ group		near to benzimidazolyl moiety
**PHE738**	**H bond with ester C=O**	near to pyrimidinyl ring and the amidic carbonyl of chain a	near to benzimidazolyl moiety
ASP801 negatively charged (-COO-)	near to pyrimidinyl ring		
HIS806	near to pyrimidinyl ring		

PRO809	near to $COOCH_3$ group	near to pyrimidinyl ring	near to benzimidazolyl moiety
LEU810		near to pyrimidinyl ring	near to benzimidazolyl moiety
GLY841		near to $COOCH_3$ group belonging to chain a	near to $COOCH_3$ group
GLY842	near to pyrimidinyl ring		near to $COOCH_3$ group
HIS843	near to pyrimidinyl ring	near to aliphatic chain a	near to aliphatic chain
ZN101	near to oxygen atom of ester group	near to oxygen atom of amide group	near to oxygen atom of amide group

a. Virtual interactions within 5 Å of chain b with: ASP626 (amidic C=O), HIS541, PRO273, and MET274 ($COOCH_3$ group)

Table S3. Hydrogen bonds and virtual interactions within 5 Å between compound **5a**, **6**, and **8a** and aminoacid residues (and zinc ion) into the binding site of HDAC2-4LXZ (PDB ID: 4LXZ) obtained from docking evaluation.

**HDAC2-4LXZ aminoacid residues in the active site**	**5a**	**6**	**8a**
ASP04		near to oxygen atom of amide group of chain a	
HIS33		near to $COOCH_3$ group of chain a	
PRO34		near to $COOCH_3$ group of chain a	
MET35			near to benzimidazolyl ring
LEU144			near to benzimidazolyl ring
HIS145	near to $COOCH_3$ group	near to $COOCH_3$ group of chain a	near to benzimidazolyl ring
HIS146	near to $COOCH_3$ group	near to $COOCH_3$ group of chain a	near to benzimidazolyl ring
GLY154	near to $COOCH_3$ group	near to $COOCH_3$ group of chain a	near to benzimidazolyl ring
PHE155		near to aliphatic chain a	near to $COOCH_3$ group
CYS156			near to benzimidazolyl ring
ASP181			near to benzimidazolyl ring
**HIS183**	**H bond with amidic C=O**	near to aliphatic chain a	**H bond with ester C=O**
TYR209	near to pyrimidinyl ring		
PHE210	near to amide C=O	near to pyrimidinyl ring	**H bond with ester C=O**
ASP269	near to $COOCH_3$ group	near to $COOCH_3$ group of chain b	

LEU276	near to aliphatic chain	near to $COOCH_3$ group of chain b	near to benzimidazolyl ring
GLY305			near to benzimidazolyl ring
GLY306		near to $COOCH_3$ group of chain b	near to benzimidazolyl ring
**TYR308**	**H bond with ester C=O**	**H bond with ester C=O**	
Zn 401	near to $COOCH_3$ group	near to $COOCH_3$ group	near to nitrogen atoms of benzimidazolyl ring
